# Psychosocial Interventions for Families with Parental Cancer and Barriers and Facilitators to Implementation and Use – A Systematic Review

**DOI:** 10.1371/journal.pone.0156967

**Published:** 2016-06-08

**Authors:** Laura Inhestern, Anne-Catherine Haller, Olga Wlodarczyk, Corinna Bergelt

**Affiliations:** 1 University Medical Center Hamburg-Eppendorf, Department of Medical Psychology, Hamburg, Germany; 2 University Medical Center Hamburg-Eppendorf, Department of Child and Adolescent Psychiatry and Psychotherapy, Hamburg, Germany; Cardiff University, UNITED KINGDOM

## Abstract

**Background:**

Parental cancer has a significant impact on minor children and families. Psychosocial interventions for affected families can provide support where necessary. This systematic review aims at providing an overview of existing interventions and support programs and focuses on the systematic investigation of barriers and facilitators for using psychosocial interventions for families affected by parental cancer (PROSPERO; registration number CRD42014013020).

**Methods:**

A search of five electronic databases (EMBASE, MEDLINE, PsycInfo, Psyndex, CINAHL) was conducted in June 2014, and updated in September 2015. We included any kind of studies reporting psychosocial support services or interventions for families affected by parental cancer. Study quality was assessed using the Mixed Method Assessment Tool. Narrative synthesis and thematic analyses were undertaken to examine the included interventions and to identify barriers and facilitators for use and implementation.

**Results:**

A total of 36 studies covering 19 interventions and support services were included in the systematic review. Interventions focused on children, parents or several family members and analyses revealed a broad picture of theoretical background and primary aims. Several studies focused on developmental or implementation phases or descriptions of interventions. Other included studies reported results of evaluations using qualitative and quantitative methods. Results suggest that interventions are helpful and that participants improved in various outcomes. The thematic analyses indicate that barriers for use of support services refer to aspects concerning the patients and families, such as practical difficulties, perceived need for support or fear of stigma. Cancer patients who understood the need and benefit of support services may have used them more often. Additionally, intervention characteristics such as a flexible structure and accessibility were important to reach families affected by parental cancer. Disease characteristics and complications in collaborations were identified as potential barriers. The provision of information about interventions by clinicians and understanding the support as part of routine care seem to be key issues for implementation and use of psychosocial support.

**Conclusion:**

This review identified a broad number of intervention concepts for families with minor children affected by parental cancer. Findings provide a basis for existing or future psychosocial interventions to anticipate potential barriers and facilitators to implementation and use and can help to reach a wider range of families in need for support.

## Introduction

Over the past years parental cancer and its impact on minor children and families has been in focus of research. Studies found that children have an increased risk of developing emotional and behavioral problems after a parental cancer diagnosis [1–3]. Sons and daughters of cancer patients can suffer from elevated levels of distress and fear of possible loss of a parent [3, 4]. Furthermore, children’s emotional functioning is associated with family functioning [5] and emotional functioning of parents [6]. On the other hand, parents themselves are confronted with specific emotional and practical challenges [7, 8]. Ill parents not only struggle with feelings of guilt [8] but also are worried about how to best tell the children about the diagnosis and fear missing milestones of their children’s life [9].

Psychosocial interventions are a crucial component of supportive care after a cancer diagnosis and can provide support for families with minor children affected by parental cancer [10]. Experts working in this field uniformly underline the need for psychosocial support for this patient group [11, 12]. Interventions can support parents in issues regarding open communication about cancer within the family or age-appropriate information about cancer. Moreover, parents can be supported emotionally and can be reassured in their parenting competence [13]. Interventions targeting children of cancer patients support the children in dealing with their parent’s illness [14]. In supportive groups, children of cancer patients overcome feelings of isolation and are encouraged to use positive coping styles and talk about their feelings [15, 16]. Some interventions for children focus on psychoeducational aspects of cancer and cancer treatment [17, 18]. Yet, in a population-based sample only 9% of cancer survivors reported to have used professional support for or with their families during the trajectory of the disease, whereas about 74% reported a need for psychosocial support [19]. The patient’s family background or the existence of minor children is not routinely assessed in outpatient counseling services for cancer patients [11]. An analysis of current specific psychosocial care offers for families with a parent with cancer reveals a heterogeneous field of intervention concepts with most of them being limited in financial resources [11].

Earlier reviews focusing on interventions for families affected by parental cancer presented an overview of structured interventions targeted at the children [14] and an overview of structured interventions for families of palliative patients [20]. A third review focused on intervention concepts for mental health support for children of parents with somatic illness [21]. The findings of these reviews suggest that there is a lack of valid and evaluated interventions for affected parents and their children.

The previous systematic reviews found that in most intervention only a small number of families were included [14, 20]. Similarly, clinicians working with affected families reported that there was a mainly positive response to offering psychosocial support, but a quite limited number of families were actually seeking support [11]. These observations suggest that acceptance and implementation of support offers may be insufficient or ineffective.

Understanding the factors that influence the use of psychosocial interventions for families with parental cancer could provide valuable information for the implementation of an intervention. Compact but clear information could help to reach a wider range of families.

To investigate the factors associated with the implementation and use of psychosocial care in families with parental cancer, we first wanted to gain an overview of the interventions and support services for parents with cancer and their minor children. Therefore, concepts and impact of interventions were examined in a systematical review. However, our focus was to systematically identify barriers and facilitators for use and implementation of psychosocial interventions for families with minor children affected by parental cancer. Based on our findings, we aimed to generate recommendations to successfully transfer intervention concepts into clinical practice.

## Method

To develop the review we used the PRISMA (Preferred Reporting Items for Systematic Reviews and Meta-Analyses) guidelines [22] (S1 Table). The PRISMA statements focus on the evaluation of intervention studies. However, we also included studies reporting development or implementation processes of interventions. The review protocol was published in the International prospective register of systematic reviews (PROSPERO; registration number CRD42014013020, S1 File).

### Search strategy

We searched the databases CINAHL, Embase, Medline, PsycInfo and Psyndex up to June 2014 with an update in September 2015. A list of key words was developed by the research team in cooperation with a librarian of the academic library of our university medical centre. The list included the following terms: (cancer or neoplasm), (parent* or child* or famil*), (intervention or counsel* or therap*) and (psychosocial or psychological) (S1 Text). We also conducted a systematic search of citations and references of included papers to identify further relevant studies and included articles of earlier relevant systematic reviews [14, 20, 21].

### Eligibility criteria

Studies were included when they met the following criteria: accessibility of full text, published in a peer-reviewed journal, language English or German, cancer patients with minor children/minor children of cancer patients, focus of article on structured psychosocial interventions for families with minor children affected by parental cancer, focus of intervention on treatment or prevention of negative psychosocial consequences. Interventions focused on single family members (children, parents, spouses) or the whole family.

We included studies reporting exclusively on development, feasibility or implementation of interventions and evaluation studies. Intervention studies were excluded when the focus of the article was on medical treatment, when interventions addressed families affected by childhood cancer or adult children, when intervention focuses on partnership, primary mental illness, health behaviour or bereavement. Moreover, studies were excluded when interventions were not structured.

### Study selection

**Fig 1:**
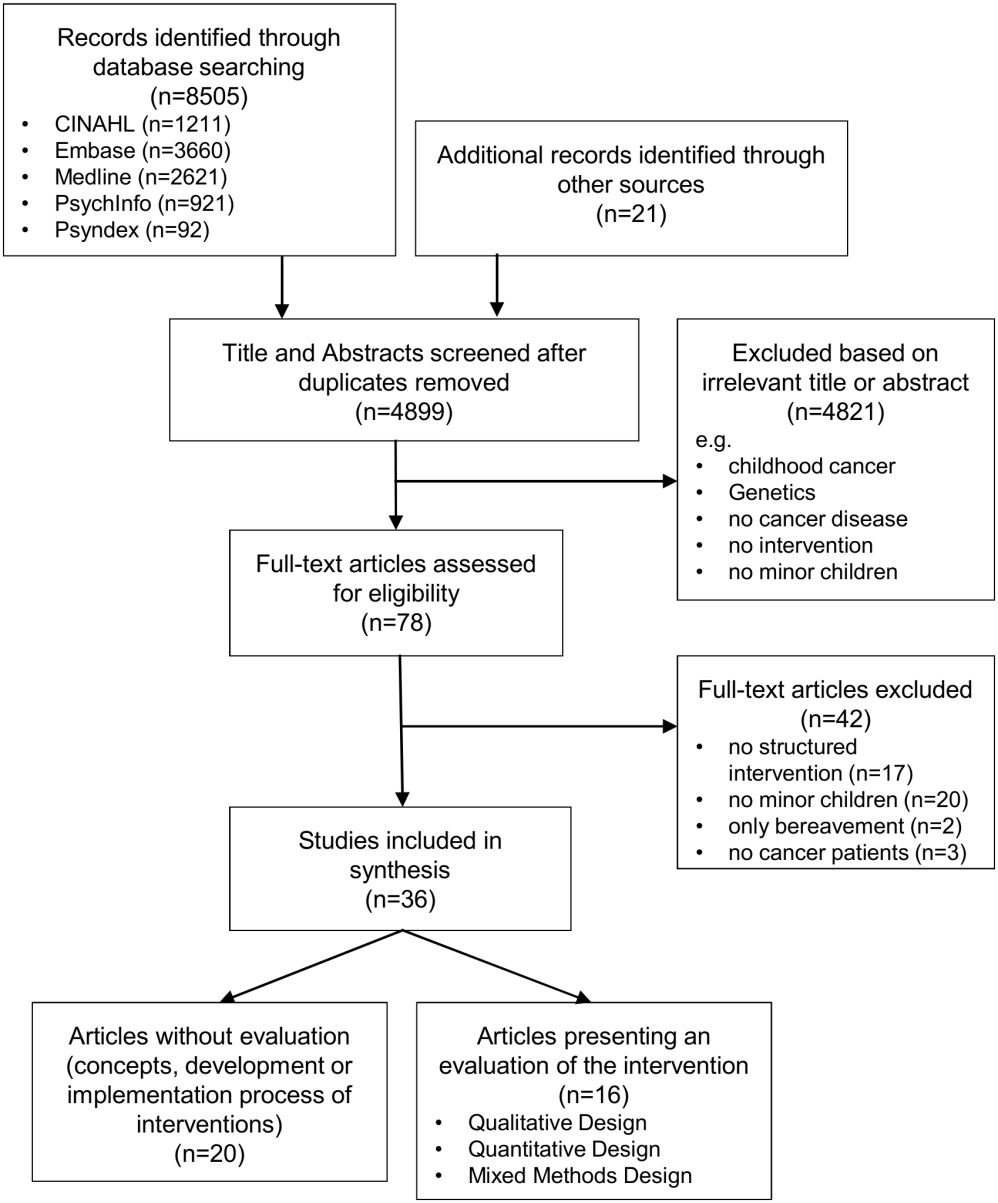
Flow diagram of systematic literature search.

Titles and abstracts of the studies identified by the searches were screened according to the predefined inclusion and exclusion criteria by two independent raters (LI, BH). When title and abstract were relevant or when eligibility was unclear the full text was retrieved. Identified full texts were screened by two reviewers (LI, ACH.) with a screening form according to the predefined inclusion and exclusion criteria (S2 Table). Any uncertainty about eligibility after assessing the full text was resolved by discussion. The flow diagram of the selection process is outlined in Fig 1.

### Data extraction and quality assessment

Included studies were classified by focus of study: (1) focus on implementation or development of the intervention and (2) evaluation study (qualitative, quantitative or mixed method design). Two of the authors (LI and ACH or OW) independently extracted data from each of the included studies using a data extraction form, which included the following information: citation details, contents of intervention, structure of intervention, way of recruitment and, if applicable, study design, study population and impact of the intervention. Barriers and facilitators for using the intervention were extracted, if reported in the paper.

To assess the quality of the included studies two reviewers (LI and ACH or OW) independently rated the studies using the Mixed Method Appraisal Tool (MMAT). The MMAT is widely used and has been demonstrated to be a reliable and valid tool assessing the methodological quality of studies with different study designs [23]. The MMAT was only considered for the evaluation studies, but not for the studies that focussed on the development or implementation phase of the intervention. In the latter no quality assessment was conducted, because studies were considered to be too heterogeneous and varied widely in content and focus. The assessment of the quality of the evaluative studies revealed an acceptable interrater-agreement of 75% and higher for the ratings on each methodological approach. Kappa values are acceptable with a range from 0.400 to 0.667. Any disagreements were solved by re-consulting the article and discussion between raters.

### Analysis Strategy

We performed a narrative analysis to synthesize the data extracted from the included studies [24]. To identify barriers and facilitators for using psychosocial intervention, we conducted an inductive thematic synthesis [25, 26].

## Results

### Included studies

The search strategy yielded a total of 8505 records with 3626 being duplicates. The secondary search identified another 21 records. Following the screening of titles and abstracts, we selected 78 potentially eligible records matching the inclusion criteria. After examination of the full texts, a total of 36 articles were included (Fig 1). These articles covered a total of 19 interventions.

Of all included records, 16 reported any kind of evaluation (evaluation study; n = 6 qualitative, n = 9 quantitative, n = 1 mixed methods), whereas 20 articles focused on the development or feasibility and implementation of interventions or described the intervention. Of these, three studies additionally described case reports and five studies reported feedback of participants of the intervention.

The methodological quality of the included evaluation studies was heterogeneous and overall could be rated as moderate. Most of them met two or three out of the four quality criteria according the MMAT (S3 Table).

### Intervention characteristics

We identified eight interventions involving parents and children [15, 27–44], seven child-centred interventions [16, 18, 45–49] and four interventions mainly focussing on working with parents [13, 50–58]. The major part of the interventions was conducted in the USA or in Europe. One intervention was conducted in Australia, one in Israel.

Most interventions focused on any kind of parental cancer (Table 1). Two interventions included only breast cancer patients [13, 27, 28, 50–52] and three interventions focused on terminal cancer [15, 31, 32, 40, 54, 55, 57]. The target group was mostly families with school-aged children. In two interventions younger children were included [38, 49]. Interventions also differed regarding their setting. Whereas some interventions were held in a group format with several families, children or parents, other interventions focused on meetings with single families or family members (Table 1). All interventions were conducted in a defined number of sessions (between 1 and 22). Only one intervention was a 3-week inpatient rehabilitation program and reported no exact number of sessions [27, 28].

**Table 1:** Summary of the included records (N = 36 studies on N = 19 different interventions).

**Name of Intervention**	**Study, Country**	**Focus of article**	**1. Diagnosis; 2. Target group**	**1. Setting; 2. Structure**	**Aims of intervention**
**Family interventions (n = 18 studies on n = 8 interventions)**					
‘Getting well together’	John et al., 2010, 2013; Germany [27, 28]	Evaluation: Quantitative design (Within-subject control group (N = 116))	1. Breast cancer; 2. Mothers and their children	1. Mother- child inpatient rehabilitation program; 2. 3 weeks	Support the family system, prevent at risk children from developing serious emotional and behavioural problems
Culturally adapted family intervention	Davey et al., 2012; USA [29]	Description/ Implementation process	1. Cancer, stage I,II,III; 2. African American families with school-aged children	1. Children's support group, multiple group family therapy; 2. 3x 90 minutes (children), 2x 120 minutes (family)	Improve family communication, improve parent-child attachment for African American families
	Davey et al., 2013; USA [30]	Evaluation: Quantitative design (IG (n = 7) vs. CG (n = 5))			
Family Focused Grief Therapy	Kissane et al., 2006, 2007; Australia [31, 40]	Evaluation: Quantitative design (IG (n = 53) vs. CG (n = 28); baseline, 6 and 13 months post bereavement)	1. Terminal cancer; 2. At risk families (based on FRI), child >12 years	1. Whole family; 2. 4–8 sessions before and after death, 90 minutes	Optimize cohesion, communication, and handling of conflict, promote the sharing of grief and mutual support
The Family Support Program (Family Talks in Cancer Care)	Bugge et al., 2008, 2009; Norway [15, 32]	Evaluation: Qualitative design (N = 6 families) up to 6 weeks after conclusion	1. Incurable cancer; 2. Whole family, children 5–18 years	1. Child, parent, family setting; 2. 5 weekly sessions	Prevent psychosocial problems, promote coping, help to talk about disease, knowledge and information about disease, help to plan for the future
Preventive Counselling Service (COSIP), Germany	Koch et al., 2011 [35]	Description of indication for intervention	1. Cancer; 2. Whole family (child <18 years)	1. Child-, parent-, family-, single setting; 2. Initial diagnostic phase, 3–8 intervention sessions	*Family level*: facilitate open communication about disease, enable flexible handling of divergent needs, prevent children from dysfunctional parentification; *Parental level*:enhance self-perceived competence in parenting, increase parental emotional availability; *Child level*:enhance cognitive comprehension of disease, legitimate individual feelings and needs, enhance active coping, integrate ambivalent feelings toward ill parent, initiate anticipatory grief
	Komo-Lang et al., 2010 [43]	Description of intervention, Case report			
	Kühne et al., 2013 [44]	Implementation process			
	Romer et al., 2007 [34]	Implementation process			
	Romer et al., 2011 [33]	Description of intervention			
	Paschen et al., 2007; Germany [36]	Evaluation: Quantitative design (post intervention (N = 25 families))	1. Somatic illness; 2. Whole family (child <18 years)		See Romer et al., 2007; *Additionally*:support parents’ use of network, enhance child’s attention to resources
Preventive Counselling Service (COSIP), Finland	Schmitt et al., 2007; Finland [41]	Developmental Phase/ implementation	1. Cancer; 2. Whole family (children <18 years)	1. Child, parent, family setting; 2. 5–6 sessions (1-2x family, 1-2x couple, 1 sibling session, 1 session each child)	Support parenting and parenthood, assess need of all family members, accompany family members in process through loss and grief
Preventive Counselling Service (COSIP), Denmark	Thastum et al., 2006; Denmark [37]	Evaluation: Qualitative and quantitative design (N = 24 families)	1. Cancer; 2. Whole family (children 8–15 years)	1. Child-, parent-, family-centred, single setting; 2. 5–6 sessions	See Romer et al. 2007; *Additionally*: increase attention in role changes, support parents in age-appropriate communication, support parents’ use of network, support parents working through mutual problems caused by disease, enhance child’s attention to resources
Specification of COSIP	Dörr et al., 2012; Germany [38]	Description of intervention, Case report	1. Cancer; 2. Families with toddlers 0–5 years	1. Parent-child-sessions; 2. Initial session, counselling sessions, final session	Support for parent-child-dyads, psychoeducation of parents, maintaining parental competence
Short-term psycho-educational intervention	Hoke, 1997; USA [42]	Description of intervention, Case report	1. Cancer; 2. Whole family	1. Child-, parent-, family-sessions; 2. About 6 sessions	Share concerns and talk about disease; increase understanding and support within families
Struggle for Life trial	Niemelä et al., 2012; Finland [39]	Evaluation: Quantitative design (Baseline, post intervention (4,10,18 months after completion), N = 19)	1. Cancer; 2. Families with children 8–17 years	1. Child-, parent-centred and family sessions; 2. 2 interventions: Let’s Talk = 2 sessions; Family Talks = 6–8 sessions	*Let’s talk*: strengthen children; *Family Talks*: support family communication, support children’s and parents’ psychosocial well-being
**Parent-centred interventions (n = 10 studies on n = 4 interventions)**					
The Enhancing Connection Program	Lewis et al., 2006; USA [50]	Evaluation (Pilot study): Quantitative design (pre-post, N = 13)	1. Breast cancer; 2. Mothers with school-aged children	1. Parent-centred, home-based sessions; 2. 5 sessions, 60 minutes	Enhance communication, decrease maternal depressed mood, improve parenting behaviour, improve children's adjustment
	Davis Kirsch et al., 2003; USA [51]	Evaluation (Pilot study): Qualitative Design (N = 4 families)			Enhance interaction between mother and child dyads
	Brandt et al., 2004; USA [52]	Implementation (Pilot study) (N = 8)			Improve the quality of mother-child relationship, improve parenting behaviour, improve children's adjustment
	Lewis et al., 2015; USA [13]	Evaluation: Quantitative design (IG (n = 90) vs. CG (n = 86) (baseline, post, follow-up))			Decrease maternal depressed mood, improve parenting behaviour, improve children's adjustment
Art-therapy program for parents	Weiß et al. 2005; Germany [58]	Description/ Development	1. Cancer; 2. Younger patients with and without children	1. Group setting; 2. 22 weekly sessions à 90 minutes	Creating something, reassure self-confidence, design a book to support communication with children
Being a parent and coping with cancer	Hasson-Ohayon & Braun, 2011; Israel [53]	Description/ Development, Feedback	1. Cancer; 2. Patients with children undergoing chemo	1. Parent-centred group; 2. 4 sessions/ 1 day workshop	Empower the patient and spouse in their parenting, help the parents to help their children to adjust and cope
Preventive Intervention for Bereaved Children	Christ et al., 1991; USA [55]	Description	1. Terminal cancer; 2. Children 7–17 years, healthy parent	1. Parent-centred (healthy) and sessions with children; 2. 6–8 sessions before death, 6–8 sessions after death, 90 minutes	Facilitate children’s adjustment to disease /death, support the well parent to deal with own grief, support continuance of well parent's parental functioning
	Christ et al., 2005; USA [56]	Evaluation: Quantitative design (IG (n = 79) vs. CG (n = 25) (pre, post, follow-up))			Support continuance of well parent's parental functioning, provide safe environment for the children
	Christ & Siegel, 1991; USA [54]	Development/ Description of intervention			Prevent deleterious effects of terminal disease and death on children and well parent
	Siegel et al., 1990; USA [57]	Development/ Description of intervention			Support the well parent to deal with own grief, support continuance of well parent's parental functioning
**Child-centred interventions (n = 7 studies on n = 7 interventions)**					
The Bear Essential Program	Greening, 1992; USA [49]	Description/ Implementation, Feedback	1. Cancer; 2. Children 4–8 years and parents	1. Child-centred and parent groups; 2. Monthly, 90 minutes	Support families in understanding each other and coping; provide supportive environment to discuss concerns
For kids only	Bedway & Smith, 1997; USA [45]	Description, Feedback/ Comments	1. Cancer; 2. Children (preschool-adolescents)	1. Child-centred group; 2. 1-day workshop	Education, support & screening of children; provide safe environment
School-based support group	Call, 1990; USA [46]	Description and Implementation	1. Cancer; 2. School-aged children 6–12 years	1. Child-centred group; 2. 10 weekly sessions, 50–55 minutes	Develop coping skills, create safe environment, share feelings, keep on with activities, educate about disease
Quest	Heiney & Lesesne, 1996; USA [47]	Description, Feedback	1. Cancer; 2. Children 5–18 years	1. Child-centred group; 2. One parent pre-program interview; 1x 2 hours, biannually	Facilitate positive coping, increase understanding about cancer and treatment, promote positive communication about diagnosis within the family system
On Belay	Tucker et al., 2013; USA [16]	Description, Evaluation: Qualitative design (Focus group with parents (n = 9) and children (n = 12))	1. Cancer; 2. Children 9–19 years	1. Child-centred, group; 2. 1 day, 8 hours	Build community among children, help children to discover personal power
Kids can cope	Taylor-Brown, 1993; Canada [18]	Description/ Development	1. Cancer; 2. Children 5–18 years	1. Child-centred group; 2. 6 weekly sessions + information session for parents	Educate children about cancer, provide a supportive environment, increase coping skills
CLIMB (Children’s lives include moments of bravery)	Semple & McCaughan 2013; Ireland, UK [48]	Evaluation: Qualitative design (interviews/focus group (n = 4 parents, n = 7 children))	1. Cancer; 2. Children 5–12 years	1. Child-centred group setting; 2. 6 weekly sessions á 90 minutes	Provide education about cancer, normalize emotions that a child experiences, support communication of emotions, improve coping
**Name of Intervention**	**Study, Country**	**Focus of article**	**1. Diagnosis; 2. Target group**	**1. Setting; 2. Structure**	**Aims of intervention**
**Family interventions (n = 18 studies on n = 8 interventions)**					
‘Getting well together’	John et al., 2010, 2013; Germany [27, 28]	Evaluation: Quantitative design (Within-subject control group (N = 116))	1. Breast cancer; 2. Mothers and their children	1. Mother- child inpatient rehabilitation program; 2. 3 weeks	Support the family system, prevent at risk children from developing serious emotional and behavioural problems
Culturally adapted family intervention	Davey et al., 2012; USA [29]	Description/ Implementation process	1. Cancer, stage I,II,III; 2. African American families with school-aged children	1. Children's support group, multiple group family therapy; 2. 3x 90 minutes (children), 2x 120 minutes (family)	Improve family communication, improve parent-child attachment for African American families
	Davey et al., 2013; USA [30]	Evaluation: Quantitative design (IG (n = 7) vs. CG (n = 5))			
Family Focused Grief Therapy	Kissane et al., 2006, 2007; Australia [31, 40]	Evaluation: Quantitative design (IG (n = 53) vs. CG (n = 28); baseline, 6 and 13 months post bereavement)	1. Terminal cancer; 2. At risk families (based on FRI), child >12 years	1. Whole family; 2. 4–8 sessions before and after death, 90 minutes	Optimize cohesion, communication, and handling of conflict, promote the sharing of grief and mutual support
The Family Support Program (Family Talks in Cancer Care)	Bugge et al., 2008, 2009; Norway [15, 32]	Evaluation: Qualitative design (N = 6 families) up to 6 weeks after conclusion	1. Incurable cancer; 2. Whole family, children 5–18 years	1. Child, parent, family setting; 2. 5 weekly sessions	Prevent psychosocial problems, promote coping, help to talk about disease, knowledge and information about disease, help to plan for the future
Preventive Counselling Service (COSIP), Germany	Koch et al., 2011 [35]	Description of indication for intervention	1. Cancer; 2. Whole family (child <18 years)	1. Child-, parent-, family-, single setting; 2. Initial diagnostic phase, 3–8 intervention sessions	*Family level*: facilitate open communication about disease, enable flexible handling of divergent needs, prevent children from dysfunctional parentification; *Parental level*:enhance self-perceived competence in parenting, increase parental emotional availability; *Child level*:enhance cognitive comprehension of disease, legitimate individual feelings and needs, enhance active coping, integrate ambivalent feelings toward ill parent, initiate anticipatory grief
	Komo-Lang et al., 2010 [43]	Description of intervention, Case report			
	Kühne et al., 2013 [44]	Implementation process			
	Romer et al., 2007 [34]	Implementation process			
	Romer et al., 2011 [33]	Description of intervention			
	Paschen et al., 2007; Germany [36]	Evaluation: Quantitative design (post intervention (N = 25 families))	1. Somatic illness; 2. Whole family (child <18 years)		See Romer et al., 2007; *Additionally*:support parents’ use of network, enhance child’s attention to resources
Preventive Counselling Service (COSIP), Finland	Schmitt et al., 2007; Finland [41]	Developmental Phase/ implementation	1. Cancer; 2. Whole family (children <18 years)	1. Child, parent, family setting; 2. 5–6 sessions (1-2x family, 1-2x couple, 1 sibling session, 1 session each child)	Support parenting and parenthood, assess need of all family members, accompany family members in process through loss and grief
Preventive Counselling Service (COSIP), Denmark	Thastum et al., 2006; Denmark [37]	Evaluation: Qualitative and quantitative design (N = 24 families)	1. Cancer; 2. Whole family (children 8–15 years)	1. Child-, parent-, family-centred, single setting; 2. 5–6 sessions	See Romer et al. 2007; *Additionally*: increase attention in role changes, support parents in age-appropriate communication, support parents’ use of network, support parents working through mutual problems caused by disease, enhance child’s attention to resources
Specification of COSIP	Dörr et al., 2012; Germany [38]	Description of intervention, Case report	1. Cancer; 2. Families with toddlers 0–5 years	1. Parent-child-sessions; 2. Initial session, counselling sessions, final session	Support for parent-child-dyads, psychoeducation of parents, maintaining parental competence
Short-term psycho-educational intervention	Hoke, 1997; USA [42]	Description of intervention, Case report	1. Cancer; 2. Whole family	1. Child-, parent-, family-sessions; 2. About 6 sessions	Share concerns and talk about disease; increase understanding and support within families
Struggle for Life trial	Niemelä et al., 2012; Finland [39]	Evaluation: Quantitative design (Baseline, post intervention (4,10,18 months after completion), N = 19)	1. Cancer; 2. Families with children 8–17 years	1. Child-, parent-centred and family sessions; 2. 2 interventions: Let’s Talk = 2 sessions; Family Talks = 6–8 sessions	*Let’s talk*: strengthen children; *Family Talks*: support family communication, support children’s and parents’ psychosocial well-being
**Parent-centred interventions (n = 10 studies on n = 4 interventions)**					
The Enhancing Connection Program	Lewis et al., 2006; USA [50]	Evaluation (Pilot study): Quantitative design (pre-post, N = 13)	1. Breast cancer; 2. Mothers with school-aged children	1. Parent-centred, home-based sessions; 2. 5 sessions, 60 minutes	Enhance communication, decrease maternal depressed mood, improve parenting behaviour, improve children's adjustment
	Davis Kirsch et al., 2003; USA [51]	Evaluation (Pilot study): Qualitative Design (N = 4 families)			Enhance interaction between mother and child dyads
	Brandt et al., 2004; USA [52]	Implementation (Pilot study) (N = 8)			Improve the quality of mother-child relationship, improve parenting behaviour, improve children's adjustment
	Lewis et al., 2015; USA [13]	Evaluation: Quantitative design (IG (n = 90) vs. CG (n = 86) (baseline, post, follow-up))			Decrease maternal depressed mood, improve parenting behaviour, improve children's adjustment
Art-therapy program for parents	Weiß et al. 2005; Germany [58]	Description/ Development	1. Cancer; 2. Younger patients with and without children	1. Group setting; 2. 22 weekly sessions à 90 minutes	Creating something, reassure self-confidence, design a book to support communication with children
Being a parent and coping with cancer	Hasson-Ohayon & Braun, 2011; Israel [53]	Description/ Development, Feedback	1. Cancer; 2. Patients with children undergoing chemo	1. Parent-centred group; 2. 4 sessions/ 1 day workshop	Empower the patient and spouse in their parenting, help the parents to help their children to adjust and cope
Preventive Intervention for Bereaved Children	Christ et al., 1991; USA [55]	Description	1. Terminal cancer; 2. Children 7–17 years, healthy parent	1. Parent-centred (healthy) and sessions with children; 2. 6–8 sessions before death, 6–8 sessions after death, 90 minutes	Facilitate children’s adjustment to disease /death, support the well parent to deal with own grief, support continuance of well parent's parental functioning
	Christ et al., 2005; USA [56]	Evaluation: Quantitative design (IG (n = 79) vs. CG (n = 25) (pre, post, follow-up))			Support continuance of well parent's parental functioning, provide safe environment for the children
	Christ & Siegel, 1991; USA [54]	Development/ Description of intervention			Prevent deleterious effects of terminal disease and death on children and well parent
	Siegel et al., 1990; USA [57]	Development/ Description of intervention			Support the well parent to deal with own grief, support continuance of well parent's parental functioning
**Child-centred interventions (n = 7 studies on n = 7 interventions)**					
The Bear Essential Program	Greening, 1992; USA [49]	Description/ Implementation, Feedback	1. Cancer; 2. Children 4–8 years and parents	1. Child-centred and parent groups; 2. Monthly, 90 minutes	Support families in understanding each other and coping; provide supportive environment to discuss concerns
For kids only	Bedway & Smith, 1997; USA [45]	Description, Feedback/ Comments	1. Cancer; 2. Children (preschool-adolescents)	1. Child-centred group; 2. 1-day workshop	Education, support & screening of children; provide safe environment
School-based support group	Call, 1990; USA [46]	Description and Implementation	1. Cancer; 2. School-aged children 6–12 years	1. Child-centred group; 2. 10 weekly sessions, 50–55 minutes	Develop coping skills, create safe environment, share feelings, keep on with activities, educate about disease
Quest	Heiney & Lesesne, 1996; USA [47]	Description, Feedback	1. Cancer; 2. Children 5–18 years	1. Child-centred group; 2. One parent pre-program interview; 1x 2 hours, biannually	Facilitate positive coping, increase understanding about cancer and treatment, promote positive communication about diagnosis within the family system
On Belay	Tucker et al., 2013; USA [16]	Description, Evaluation: Qualitative design (Focus group with parents (n = 9) and children (n = 12))	1. Cancer; 2. Children 9–19 years	1. Child-centred, group; 2. 1 day, 8 hours	Build community among children, help children to discover personal power
Kids can cope	Taylor-Brown, 1993; Canada [18]	Description/ Development	1. Cancer; 2. Children 5–18 years	1. Child-centred group; 2. 6 weekly sessions + information session for parents	Educate children about cancer, provide a supportive environment, increase coping skills
CLIMB (Children’s lives include moments of bravery)	Semple & McCaughan 2013; Ireland, UK [48]	Evaluation: Qualitative design (interviews/focus group (n = 4 parents, n = 7 children))	1. Cancer; 2. Children 5–12 years	1. Child-centred group setting; 2. 6 weekly sessions á 90 minutes	Provide education about cancer, normalize emotions that a child experiences, support communication of emotions, improve coping

IG, Intervention group; CG, Control Group; FRI, Family Relationship Index

Interventions mainly aimed at improving the communication between family members [29, 31, 33–36, 39, 42–44, 47, 50, 53] and at supporting children’s adjustment to and understanding of cancer [13, 15, 18, 32–36, 39, 43–49, 52, 54–57] (Table 1). Interventions were conducted in order to support children and families in their coping strategies [15, 18, 32–36, 47, 48, 53] and to reassure parents in their parenting skills [13, 33–38, 41, 43, 44, 52, 53]. Other aims were to improve the attachment of children and parents [38, 51], to improve parental mood [13, 39, 50, 52] or to support grieving processes [31, 33–36, 40, 43, 44, 54–57].

### Background and evaluation of the interventions

Interventions were developed based on a broad range of approaches and theoretical background (S4 Table). Some interventions were based on clinical practice and patient focus groups [29, 30, 41, 45, 46, 53–58] or on literature on children affected by parental cancer [41, 47, 54–57]. Other interventions adapted research on families with a mentally ill parent to families with somatically ill parents [15, 29, 30, 32, 39, 42]. Coping theory, social cognitive theory, attachment theory, family therapy or systematic approach were also used for the development of interventions [13, 15, 27, 28, 31–37, 40, 50–52]. Other aspects in the development of interventions included art as support and adventure-based approach [16, 58]. For one intervention only the theoretical background was not stated [18].

#### Evaluation of family interventions

The evaluation studies on family interventions reported improvements in several outcomes: One study described improvements in the quality of life of parents and children [27, 28] (S4 Table). Other studies found improvements in depression scores and psychiatric symptoms in parents [37, 39] as well as in children’s psychological health and depressive symptoms [27, 28, 37]. Other studies did not find significant changes in parent’s or children’s depression or distress scores [30, 31, 40]. In one study, the most distressed family members at the beginning of the intervention benefited the most with regard to distress and depression [31, 40]. With regard to children’s anxiety, self-concept and attachment a study by Thastum and colleagues (2006) did not find significant improvements after the intervention. In other studies, improvements in family communication and regarding several other aspects of family functioning were found [29, 30, 37].

In qualitative studies on family interventions, parents reported an increase of open communication and sharing of feelings between family members, being reassured to be a good parent and to feel a normalization of emotions [32, 37] after the intervention. Children reported to have better coping strategies and to talk more openly about the parental disease [15].

#### Evaluation of parent-centred interventions

Evaluation studies investigating the impact of parent-centred interventions reported an improvement in mother’s functioning [13, 50] and effects on parenting skills two months after the intervention [13]. However, improvements were not significant 12 months after the intervention [13]. Parents reported less emotional and behavioural problems as well as lower depressed mood in their children [13, 50]. Regarding the quality of mother-child relationship and children’s illness-related pressure, grief, anxiety and depressive symptoms no significant changes were found after the intervention [50]. In another evaluation study children reported improvements in the parenting competence and communication competence on their parents [56].

In the only qualitative evaluation study mothers reported to be more aware of their own feelings and observed an enrichment of the mother-child relationship [51]. Similarly fathers reported that children and mothers had a closer relationship after the intervention. Moreover, fathers reported changes in the children’s behaviour such as being more patient [51].

#### Evaluation of child-centred interventions

Only two—qualitative—studies reported on the evaluation of child-centred interventions. Child-centred groups helped to build a social bond between the children and to normalize the experience of having a parent with cancer [16, 48]. In the adventure-based approach children experienced how to deal with difficult situations and how to master challenges [16]. Improvements were reported in the understanding of cancer, coping strategies, mood and behaviour of the children [48].

### Barriers for using psychosocial support

**Fig 2:**
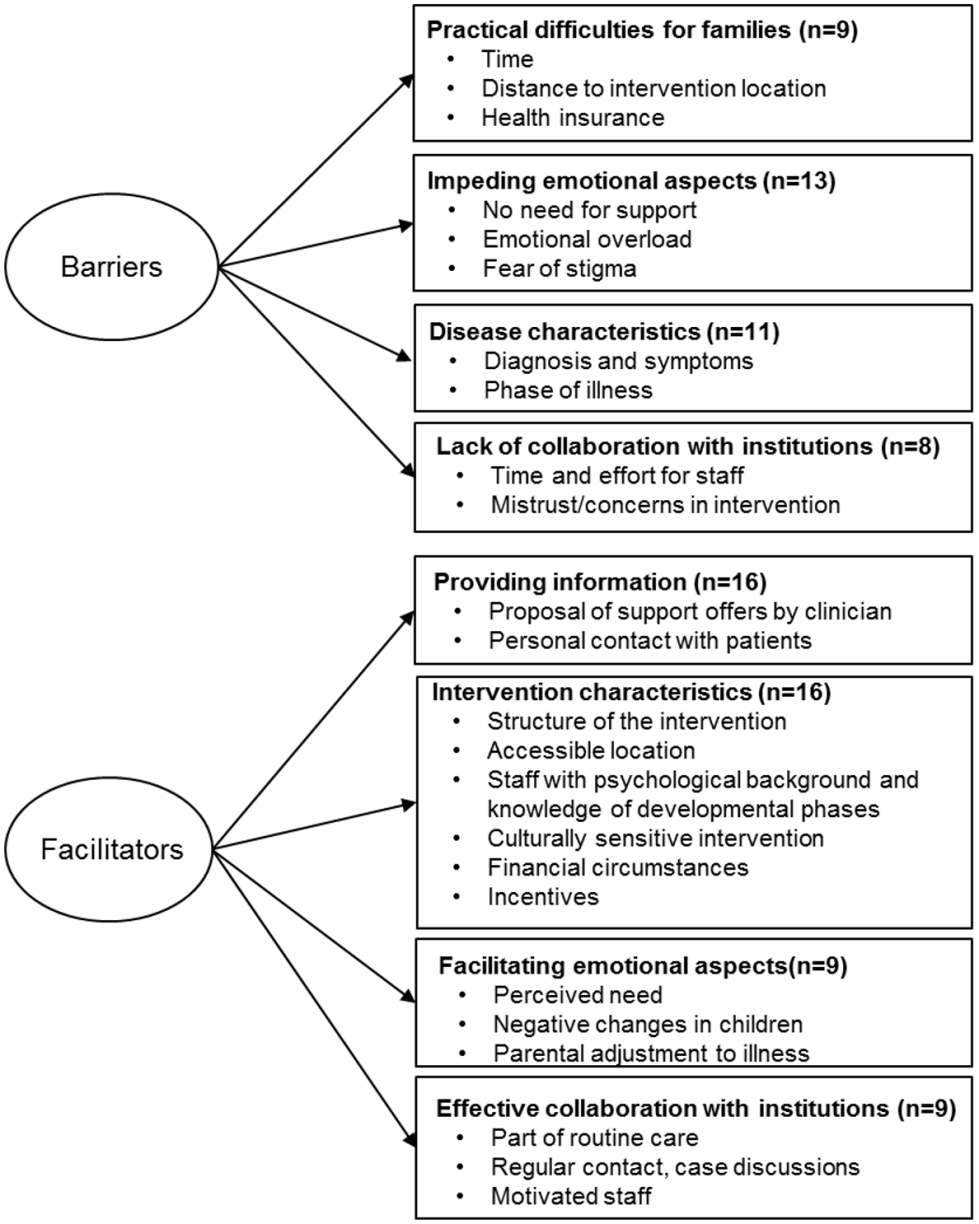
Barriers and facilitators for using psychosocial support services in families with parental cancer.

Only a few studies explicitly reported barriers and facilitators for using psychosocial support services or interventions in families affected by parental cancer. Most studies reported barriers and facilitators that where derived from the experience of the study team during the implementation processes or recruitment phases. Some studies did not report any barriers and facilitators (S5 Table). We identified four dimensions of barriers for using psychosocial support when a parent with minor children has cancer (Fig 2).

#### Practical difficulties for the families

Firstly, studies reported about practical difficulties for the families. Due to treatment and other obligations [13, 44, 53] parents had *little time* and therefore difficulties to commit to regular sessions. Moreover, *living too far from the location of the intervention* prevented families from using psychosocial support [41, 48]. Difficulties regarding the transportation of the children [18, 46] or moving to other regions [56] were also mentioned as factors reducing the likelihood of participation in interventions. One group intervention for children was conducted during school hours which led to the children needing to catch up classwork after school [46]. In an inpatient rehabilitation program for mothers and their children the approval to take part in the program depended on the *health insurance* providers [27], which was a barrier for some families.

#### Impeding emotional aspects

Another dimension of barriers referred to impeding emotional aspects of affected families. Some studies reported families had *no need* for psychosocial support [13, 31, 34, 47, 55, 56]. For example families were coping well [31] or were already in psychiatric care [41]. One study team assumed that some parents were unable to access their children’s need [47]. One study reported that in some cases at least one family member was against the participation [41]. In other studies the study team suggested that some parents refused psychosocial support to prevent themselves or their children from *emotional overload*. Parents may feel overwhelmed by the situation [34, 38, 53, 55] and may not want to confront their children with the situation of the parental disease [13, 55]. One study focusing on an inpatient rehabilitation program reported that mothers fear that time spent away from home may harm their children [27, 28]. Another study suggested that families may fear psychosocial support as an inference with their own coping [41]. *Fear of stigma* can also be a barrier for using psychosocial support [34, 38, 41, 44]. Families may have prejudices towards psychiatry [41] or mistrust psychosocial support services [29, 30].

#### Disease characteristics

Other barriers for using psychosocial support referred to the characteristics of the disease and its treatment. Due to *symptoms and progress of the disease* or treatment side effects patients may not be able to take part in any intervention [13, 15, 31, 32, 34, 41, 55, 56]. Studies reported on different *phases of disease* that would constitute a barrier. In one study in a palliative setting, patients and their families had difficulties to accept the failure of treatment [55] and therefore would not commit to the intervention. Greening (1992) reported that particularly newly diagnosed patients did not seem to be interested in taking part in the intervention. Similar observations are reported by Romer and colleagues (2007), who found that referral soon after diagnosis did not lead to use of the intervention.

#### Lack of collaboration with clinics and institutions

Additionally to barriers regarding the patients and families, the collaboration with clinics or other institutions can also be an impeding factor when implementing psychosocial support services. According to some of the studies, building networks to implement interventions needs a lot of *time and effort* of both, the study team and the staff of the institutions [29, 34, 41, 44, 46]. Moreover, in some cases physicians seemed to *mistrust the use of psychosocial support*, for example because feelings and fears of the families may have been “well repressed” [34]. Kühne et al. (2013) reported about cooperating partners who only referred acute demand cases. In an intervention regarding the palliative setting starting about six months before parental death, the study team reported difficulties with clinicians who refused foreseeing parental death [54] and who had concerns that if they did so the patient may feel they “had given up on the patient” [55].

### Facilitators for using psychosocial support

We identified four different dimensions of facilitators for using psychosocial support services (Fig 2, S5 Fig).

#### Information about support services

The most frequently mentioned facilitator for the families was the provision of information about the psychosocial support service. Many studies describe that the most promising way is when *the clinician or medical staff proposes* the support service [13, 34, 39, 41, 43, 44, 47, 49, 50, 54–56]. Following that, families are often *contacted personally* by a member of the study or intervention team [13, 15, 29, 34, 38, 43, 47, 48, 50, 54, 56].

#### Interventions characteristics

Other aspects facilitating the use of support services concern several characteristics of the interventions. First, the *structure of the intervention* seems to be an important factor. Several studies reported to have changed the originally planned structure of the intervention to adjust to the situation of their target group [45, 46, 53–55], which was appreciated by the attendees. Moreover, interventions were adapted to the needs of the families [34, 36, 43, 44] or their living situation, e.g., separated and divorced parents or new partners also being invited to participate [32]. In one intervention the team decided to offer a babysitting service for younger children to enable other family members to take part in the sessions [29, 30]. Another team decided to provide meals for participating children to overcome timing difficulties [48]. Furthermore, the *accessibility of the location* is an important factor for families to use the psychosocial support services [45]. For example, support can be provided in a location where the patient receives the medical treatment [44] or a group for children could be located at school [46]. Two interventions offered support with transportation [18, 29, 30]. Other characteristics of the interventions, which facilitate their utilization, were *staff with expertise in psycho-oncology and good communication skills with children* [15, 29, 32, 45], *cultural sensitivity* [29, 30], *safe financial circumstances of the intervention itself* [45] and offering financial *incentives* to families for participating in all sessions [29, 30].

#### Facilitating emotional aspects

In some studies patient’s and families’ emotional situation facilitated using support services. P*erceiving a need* for themselves, their children or their families was identified as a critical condition for using support services [15, 28, 32, 36, 37, 41, 47, 55]. Some studies reported that parents noticed *changes in their children’s behaviour* and therefore sought support [28, 48]. Other studies suggested that parents had to *have adjusted to their disease* and the situation themselves before seeking support for their families [34, 41].

#### Effective collaboration with clinics and institutions

Well-functioning and established collaborations with clinics and other institutions can positively impact the use of psychosocial support services in families with parental cancer. Studies reported that implementing an intervention or support service as *part of routine care* for affected families could facilitate the use of support for patients and families [29, 32, 34, 41, 44, 47]. It can be important to have a *contact person* in clinics for the interested families/patients to get in touch with or to find other ways for the interested families/patients to regularly contact the medical staff [29, 32, 34, 41, 44, 47, 56]. Moreover, the *motivation and engagement of the clinic staff* can influence the use of support services: for example, two studies reported explicitly about the implementation of an intervention in clinics which had specifically expressed the interest to establish support for affected families [15, 34].

## Discussion

This systematic review included a total of 36 studies covering 19 different interventions for families with minor children affected by parental cancer. Interventions focused on parents, children or several family members. The narrative synthesis of records revealed a heterogeneous picture of the theoretical background, primary aims and impact of the interventions.

Compared to previous systematic reviews on interventions for families affected by parental disease [14, 20, 21], our study included parent- and child-centred interventions as well as interventions including various combinations of family members when a parent has cancer. We found some intervention concepts that were covered by several publications, such as the work of the COSIP (children of somatically ill parents) group [33–37, 41, 43, 44], the enhancing connections program [13, 50–52] or a preventive intervention for bereaved children [54–56]. Other interventions were only represented by single publications (Table 1). Many articles reported only a description of the intervention without any evaluation. Only a few articles reported evaluative results including a control group [30, 56] or the use of a randomized controlled design [13, 31]. We found several studies using a qualitative design to evaluate the intervention. Nonetheless, most interventions were assessed to be helpful [16, 36, 54, 56] and reported improvements in quality of life [27], mental health or distress [13, 31, 37, 39]. Moreover, interventions supported the families to promote open communication in the families [15, 30, 32, 37, 48, 51] or enabled family members to better understand each other’s feelings [15, 32, 53]. In addition to parental and child’s distress or mental health, more specific measures reflecting the parental situation such as the parenting concerns questionnaire may be useful to investigate the impact of psychosocial support for affected families [59, 60].

Several studies explicitly or implicitly reported barriers and facilitators on implementation or use of psychosocial support services or interventions for affected families. Using thematic analysis, we summarized the findings and developed a schematic overview of barriers and facilitators. We identified four domains of barriers and four domains of facilitators for implementation and use of psychosocial support offers for families with parental cancer (Fig 2).

Following the framework for implementation of research by Damschroder and colleagues [61], we identified different domains influencing the implementation of support services for families affected by parental cancer. Similarly to Damschroder et al., our results suggest that characteristics of the interventions and support programs are an important factor influencing the implementation. Interventions should ideally be adaptable for particular settings (e.g. inpatient or outpatient care) and flexible regarding the situation of the families (e.g. the physical capability of the ill parent). With regard to the context of the support offer it seems promising to explain and offer the support by a clinician and after that personally contacts the families. For successful collaborations with clinics and other institutions regular contact between all actors are important and necessary. Support offers should be part of routine care and be integrated in the organisational setting of clinics or other institutions. To accomplish this, the process of implementation should follow the activities presented in the framework of implementation [61]: careful planning, engaging and involving members of teams (e.g. by training or case discussion), executing the implementation plan and reflecting and evaluating the implementation process to improve and adapt routines where necessary.

To overcome barriers for the use of psychosocial support according to the needs of affected families, our review suggests that professionals should address possible consequences of a parental disease for the parents themselves as well as for the emotional situation of the children. Moreover, it seems important to emphasize the preventive character of support offers. This may lead to a decrease of fear of stigmatization. As the studies included in our systematic review mostly referred to pilot projects or interventions conducted in a study context, questions on a particular way to overcome barriers such as distance to the support offer or physical capability of the parents remain unanswered so far. However, when developing a support service, these aspects should be anticipated and feasible ways to handle such cases flexibly should be worked out.

This systematic review has several strengths: In order to provide a broad picture of the entire field of interventions and psychosocial support services we decided that the study design or quality was not a criterion for exclusion from this review. This approach combined with a detailed and systematic search strategy which included five electronic databases led to a heterogeneous sample base of studies. Therefore, the review provides a comprehensive overview of existing interventions and aggregates the existing knowledge about barriers and facilitators for the implementation and use of support services for families affected by parental cancer. To ensure the reliability of the results, all processes of the systematic review were conducted by two reviewers independently, including screening of abstracts and titles, full text examination, data extraction and quality assessment. There are some limitations to report of: First, our search was limited to studies in English or German thereby excluding intervention concepts or support services published in other languages. We might have excluded records on mixed samples also including cancer patients which also might have been relevant. Although developing our search strategy carefully, we were not able to identify all relevant studies and thus, in a secondary research, added several studies from reference lists of studies included in the review. Most of the included studies were conducted in the USA and Europe. Hence, we cannot draw conclusions for interventions in cultures apart from western countries. Another limitation is that some studies did not report any barriers and facilitators. Yet, studies teams might have experienced factors affecting the use of psychosocial support, which could not be included in our analysis.

## Conclusion

To our knowledge, this is the first systematic review that not only summarizes existing structured psychosocial interventions and support services for families affected by parental cancer, but also identifies barriers and facilitators for implementation and use of those services and provides information for the implementation of such support offers. The results suggest that the development and the implementation of intervention programs for families with minor children and parental cancer needs careful planning of the intervention and the implementation process. Building tight collaborations as well as anticipating barriers to using the support offers is crucial components for successful implementation.

This systematic review can help existing interventions or psychosocial support services to optimize their approach and gives information for implementation of future interventions for families affected by parental cancer.

## Supporting Information

S1 File Protocol. (PDF)

S1 Table PRSIMA Checklist. (DOC)

S2 Table Inclusion and exclusion criteria. (DOCX)

S3 Table Quality assessment of articles reporting any kind of evaluation (n = 17) using the MMAT. (DOCX)

S4 Table Background and impact of interventions reported in included studies. (DOCX)

S5 Table Thematic analysis of barriers and facilitators for using psychosocial support services in families affected by parental cancer. (DOCX)

S1 Text Eletronic database search strategy for EMBASE. (DOCX)
