# Supplementary material for: Psychosocial Interventions for Families with Parental Cancer and Barriers and Facilitators to Implementation and Use – A Systematic Review
Source: PLoS One. 2016 Jun 8;11(6):e0156967. doi: 10.1371/journal.pone.0156967 (PMC4898703; doi:10.1371/journal.pone.0156967)
Supplement: S1 File — (PDF) [file pone.0156967.s001.pdf]

UNIVERSITY of York
Centre for Reviews and Dissemination

NHS
National Institute for
Health Research

PROSPERO International prospective register of systematic reviews

Psychosocial interventions for families with parental cancer and barriers and facilitators to implementation and use: a systematic review

Laura Inhestern, Anne-Catherine Haller, Corinna Bergelt, Olga Wlodarczyk

Citation

Laura Inhestern, Anne-Catherine Haller, Corinna Bergelt, Olga Wlodarczyk. Psychosocial interventions for families with parental cancer and barriers and facilitators to implementation and use: a systematic review. PROSPERO 2014:CRD42014013020 Available from
http://www.crd.york.ac.uk/PROSPERO_REBRANDING/display_record.asp?ID=CRD42014013020

Review question(s)

The aim of this review is to systematically search studies on psychosocial interventions for families with minor children affected by parental cancer.

The second aim of this review is to identify barriers and facilitators to using psychosocial interventions for these families

Searches

The following data bases will be searched:

- MEDLINE

- EMBASE

- PsycINFO

- PSYNDEX

- CINAHL

The search strategies will be adjusted to match each data base. There will be no restrictions regarding publication year. Journal articles in English and German will be included. Searches will be limited to human subjects. Additionally hand searches of references of relevant papers will be conducted.

Types of study to be included

Anticipating that there will be few studies reporting barriers to and facilitators for use of psychosocial support service in this population there are no restrictions on the type of study design.

Condition or domain being studied

The review focuses on psychosocial interventions for families with minor children affected by parental cancer and possible barriers and facilitators for using those.

Participants/ population

Studies will be included if they focus on any kind of psychosocial support services for families affected by parental cancer. Barriers and facilitators can be assessed by experts or therapists or be reported by patients/families themselves. No restrictions are made concerning the type of cancer, cancer stage or type of intervention (counseling, family-therapy, group or single setting).

Studies will be excluded if they focus on medical treatment; families with adult children or childhood cancer are addressed.

Intervention(s), exposure(s)

Studies will be included if the support services are child-focused, family-focused or parent-focused and should target families with minor children and one or both parent(s) diagnosed with cancer. Couple interventions are only included if they focus on parenting themes.

Studies will be excluded if they focus on interventions regarding partnership, on health-oriented interventions (e.g. sun protection, cancer screening) or on other non-psychosocial interventions.

Comparator(s)/ control

Not applicable.

Outcome(s)

Primary outcomes

Psychosocial interventions and barriers and facilitator to using those

Secondary outcomes

None

Data extraction, (selection and coding)

Titles and abstracts will be screened to identify studies that potentially meet the scope of our study and the inclusion criteria. The retrieved full texts will be assessed for eligibility independently by two review authors. Disagreement will be solved by consent after discussion.

References of included studies will be hand-searched to identify further relevant studies. Data extraction will be conducted with the help of a form developed for this study on basis on former extraction forms. To assure the form is appropriate to extract the information pilot testing will be conducted. Extraction will be performed by two members of our research team. Again differences will be resolved by consent after discussion.

Following information will be extracted:

a) Authors

b) Year

c) Country

d) Method

e) Kind of Intervention/Support service

f) Theoretical framework of intervention/support service

g) Effects of Intervention

h) Sample characteristics and sample size

i) Barriers to intervention/support service

j) Facilitators for using intervention/support service

Risk of bias (quality) assessment

Retrieved full texts will be assessed by two independent reviewers for methodological quality. The Mixed Methods Appraisal Tool (MMAT) designed for reviews including qualitative, quantitative and mixed methods studies will be used to appraise the quality of included studies (Pluye & Hong, 2014).

Strategy for data synthesis

Data synthesis will be conducted by combining two syntheses (quantitative and qualitative) and performing a third synthesis (Harden, 2010). Assuming that data are heterogeneous for the first synthesis (quantitative studies) a narrative approach is proposed to summarize the findings (Dixon-Woods et al., 2005). In the second synthesis thematic analysis will be used to synthesize the findings of qualitative studies (Mays, Pope & Popay, 2005). Finally a third synthesis will be carried out to integrate the findings of qualitative and quantitative studies.

Analysis of subgroups or subsets
None planned.

Contact details for further information
Miss Inhestern

Department of Medical Psychology

University Medical Center Hamburg-Eppendorf

Martinistrasse 52

20246 Hamburg

Germany

l.inhestern@uke.de

Organisational affiliation of the review
University Medical Center Hamburg-Eppendorf, Department of Medical Psychology

www.uke.de/institute/medizinische-psychologie/

Review team
Miss Laura Inhestern, University Medical Center Hamburg-Eppendorf, Department of Medical Psychology
Miss Anne-Catherine Haller, University Medical Center Hamburg-Eppendorf, Department of Child and Adolescent Psychiatry, Psychotherapy, and Psychosomatics
Dr Corinna Bergelt, University Medical Center Hamburg-Eppendorf, Department of Medical Psychology
Miss Olga Wlodarczyk, University Medical Center Hamburg-Eppendorf, Department of Medical Psychology

Anticipated or actual start date
01 June 2014

Anticipated completion date
30 November 2015

Funding sources/sponsors
The review is part of a dissertation project in context of a junior research group funded by the Association for the Promotion of Science and Humanities in Germany (Stifterverband für die Deutsche Wissenschaft).

Conflicts of interest
None known

Language
English

Country
Germany

Subject index terms status

Subject indexing assigned by CRD

Subject index terms
Adaptation, Psychological; Child; Humans; Neoplasms; Social Support

Stage of review
Ongoing

Date of registration in PROSPERO
26 August 2014

Date of publication of this revision
01 February 2016

DOI
10.15124/CRD42014013020

Stage of review at time of this submission	Started	Completed
Preliminary searches	Yes	Yes
Piloting of the study selection process	Yes	Yes
Formal screening of search results against eligibility criteria	Yes	Yes
Data extraction	Yes	Yes
Risk of bias (quality) assessment	Yes	Yes
Data analysis	Yes	Yes

PROSPERO
International prospective register of systematic reviews
The information in this record has been provided by the named contact for this review. CRD has accepted this information in good faith and registered the review in PROSPERO. CRD bears no responsibility or liability for the content of this registration record, any associated files or external websites.
