# Supplementary material for: Psychosocial Interventions for Families with Parental Cancer and Barriers and Facilitators to Implementation and Use – A Systematic Review
Source: PLoS One. 2016 Jun 8;11(6):e0156967. doi: 10.1371/journal.pone.0156967 (PMC4898703; doi:10.1371/journal.pone.0156967)
Supplement: S2 Table — (DOCX) [file pone.0156967.s003.docx]

S2 Table: *Inclusion and exclusion criteria*

| Inclusion criteria | |
| --- | --- |
| (1) | Accessibility of full text |
| (2) | Language English or German |
| (3) | Article published in a peer-reviewed journal |
| (4) | Study population: cancer patients with minor children/ minor children of cancer patients |
| (5) | Focus of article: structured intervention/ psychosocial support service |
| (6) | Focus of intervention: treatment or prevention of negative psychosocial consequences of cancer diagnosis in parents, children, or families |
| Exclusion criteria | |
| (1) | Focus of article: medical treatments |
| (2) | Intervention addresses families with adult children or families affected by childhood cancer |
| (3) | Intervention focusses on partnership, primary mental illness or health behavior |
| (4) | Intervention focusses only on bereavement |
