# Supplementary material for: Psychosocial Interventions for Families with Parental Cancer and Barriers and Facilitators to Implementation and Use – A Systematic Review
Source: PLoS One. 2016 Jun 8;11(6):e0156967. doi: 10.1371/journal.pone.0156967 (PMC4898703; doi:10.1371/journal.pone.0156967)
Supplement: S3 Table — (DOCX) [file pone.0156967.s004.docx]

S3 Table: Quality assessment of articles reporting any kind of evaluation (n=17) using the MMAT

| References | Methodological approach | Appraisal of quality | | | |
| --- | --- | --- | --- | --- | --- |
|  | 1. Qualitative | **1.1 Are the sources of qualitative data relevant to address the research question?** | **1.2. Is the process for analyzing qualitative data relevant to address the research question (objective)?** | **1.3. Is appropriate consideration given to how findings relate to the context, e.g., the setting, in which the data were collected?** | **1.4. Is appropriate consideration given to how findings relate to researchers’ influence, e.g., through their interactions with participants?** |
| Brandt et al., 2004 |  | Yes | Yes | Unclear | Unclear |
| Bugge et al., 2008 |  | Yes | Yes | No | Yes |
| Bugge et al., 2009 |  | Yes | Yes | No | No |
| Davis Kirsch et al., 2003 |  | Yes | Yes | Unclear | Yes |
| Semple et al., 2013 |  | Yes | Unclear | Yes | Yes |
| Thastum et al. 2006 |  | Yes | Yes | Yes | Yes |
| Tucker et al., 2013 |  | Yes | Yes | Unclear | Yes |
|  | 2. Quantitative randomized controlled | **2.1. Is there a clear description of the randomization (or an appropriate sequence generation)?** | **2.2. Is there a clear description of the allocation concealment (or blinding when applicable)?** | **2.3. Are there complete outcome data (80% or above)?** | **2.4. Is there low withdrawal/drop-out (below 20%)?** |
| Christ et al., 2005 |  | No | No | No | Yes |
| Kissane et al., 2006 |  | Yes | Yes | Yes | Yes |
| Kissane et al., 2007 |  | Unclear | Unclear | Unclear | Yes |
| Lewis et al., 2014 |  | Yes | Yes | Yes | No |
|  | 3. Quantitative non-randomized | **3.1. Are participants (organizations) recruited in a way that minimizes selection bias?** | **3.2. Are measurements appropriate regarding the exposure/ intervention and outcomes?** | **3.3. In the groups being compared are the participants comparable, or do researchers take into account (control for) the difference between these groups?** | **3.4. Are there complete outcome data (80% or above), and, when applicable, an acceptable response rate (60% or above), or an acceptable follow-up rate for cohort studies (depending on the duration of follow-up)?** |
| Davey et al., 2013 |  | Yes | Yes | Yes | Unclear |
| John et al., 2010 |  | Yes | Unclear | Yes | Yes |
| John et al., 2013 |  | Yes | Yes | Yes | No |
|  | 4. Quantitative descriptive | **4.1. Is the sampling strategy relevant to address the quantitative research question?** | **4.2. Is the sample representative of the population understudy?** | **4.3. Are measurements appropriate (clear origin, or validity known, or standard instrument)?** | **4.4. Is there an acceptable response rate (60% or above)?** |
| Lewis et al., 2006 |  | Yes | Unclear | Yes | Unclear |
| Niemelä et al., 2012 |  | Yes | No | Yes | No |
| Paschen et al., 2007 |  | Yes | Unclear | Yes | No |
| Thastum et al., 2006 |  | Unclear | Yes | Yes | Yes |
|  | 5. Mixed | **5.1. Is the mixed methods research design relevant to address the qualitative and quantitative research questions (or objectives), or the**  **qualitative and quantitative aspects of the mixed methods question (or objective)?** | **5.2. Is the integration of qualitative and quantitative data (or results*) relevant to address the research question (objective)?** | **5.3. Is appropriate consideration given to the limitations associated with this integration, e.g., the divergence of qualitative and quantitative data (or results*) in a triangulation design?** | ***Criteria for the qualitative component (1.1 to 1.4), and appropriate criteria for the quantitative component (2.1 to 2.4, or 3.1 to 3.4, or 4.1 to 4.4), must be also applied.*** |
| Thastum et al., 2006 |  | Yes | Yes | Yes |  |
