# Supplementary material for: Psychosocial Interventions for Families with Parental Cancer and Barriers and Facilitators to Implementation and Use – A Systematic Review
Source: PLoS One. 2016 Jun 8;11(6):e0156967. doi: 10.1371/journal.pone.0156967 (PMC4898703; doi:10.1371/journal.pone.0156967)
Supplement: S4 Table — (DOCX) [file pone.0156967.s005.docx]

S4 Table: Background and impact of interventions reported in included studies

Table S4. *Summary of background and impact of included (N=36 records on N=19 interventions)*

| Name of Intervention | Study, Country | Focus of article | | Background theory | Impact of intervention |
| --- | --- | --- | --- | --- | --- |
| Family interventions (n=18 studies on n=8 interventions) | | | | | |
| ‘Getting well together’ | John et al., 2010; Germany (28) | Evaluation: Quantitative design (Within-subject control group (N=116)) | | Resource oriented positive psychology, stress and coping research, systemic solution focused therapy, work of the COSIP group | Higher improvement in mother’s QoL in intervention period,  Higher improvement in children’s QoL and psychological symptoms |
|  | John et al., 2013; Germany (27) |  |  |  |  |
| Culturally adapted family intervention | Davey et al. 2012; USA (29) | Description/ Implementation process | | Focus groups with African American families, Clark’s school-age child support group model, Beardslee's family therapy | Not investigated |
|  | Davey et al. 2013; USA (30) | Evaluation: Quantitative design (IG (n=7) vs. CG (n=5)) | |  | Higher improvement in family communication in intervention group, no changes in parent’s and children’s distress and view on relationships; high satisfaction with the program |
| Family Focused Grief Therapy | Kissane et al., 2006; Australia (31) | Evaluation: Quantitative design (IG (n=53) vs. CG (n=28); baseline, 6 and 13 months post bereavement) | | Classification of families into functional intermediate & dysfunctional; offering systematic approach to families at risk | No group differences in distress, bereavement phenomenology, depression and social adjustment, global family functioning; significant differences in 10% of the most distressed family members in distress and depression |
|  | Kissane et al., 2007; Australia (40) |  |  |  |  |
| The Family Support Program (Family Talks in Cancer Care) | Bugge et al., 2008; Norway (15) | Evaluation: Qualitative interviews (N=6 families) up to 6 weeks after conclusion | | Family resilience theory, coping theory (Libo & Griffith), Bearslee's preventive intervention (mental ill parent) | *Children:* coping, improved communication in family, talk about illness |
|  | Bugge et al., 2009; Norway (32) |  |  |  | *Parents:* improved family communication, support in parenting and understanding their children |
| Preventive Counselling Service (COSIP) | Koch et al., 2011; Germany (35) | Description of indication for intervention | | Developmental psychotraumatology, attachment theory, family therapy, developmental views on children’s coping | Not investigated |
|  | Komo-Lang et al., 2010; Germany (43) | Description of intervention, Case report | |  | Not investigated |
|  | Kühne et al., 2013 (44) | Implementation process | |  | Not investigated |
|  | Romer et al., 2007; Germany (34) | Implementation process | |  | Not investigated |
|  | Romer et al., 2011; Germany (33) | Description of intervention | |  | Not investigated |
|  | Paschen et al., 2007; Germany (36) | Evaluation: Quantitative design (post intervention (N=25 families)) | |  | Families evaluated counselling service positively, families were satisfied with goal achievement |
|  | Schmitt et al., 2007; Finland (41) | Developmental Phase/ implementation | | Earlier literature and clinical experiences | Not investigated |
|  | Thastum et al., 2006; Denmark (37) | Evaluation: Qualitative and quantitative design (N=24 families, before and after counselling) | | See Romer et al., 2005 | *Qualitative:* More sharing illness-related emotions, more open communication; confirmation to be a good parent; normalization of feelings;  *Quantitative:* Improvements in parent’s depression scores and in several aspects of family functioning (communication, active responsiveness, general functioning); no changes in several aspects of family functioning (roles, affective involvement, behavior control);  Improvements in children’s depression scores, no improvements in children’s self-concept, anxiety and parent and peer attachment |
| Specification of COSIP | Dörr et al., 2012; Germany (38) | Description of intervention, Case report | | Communication-based parent-child-psychotherapy, STEEP-Program, Marte-Meo-Method | Reassurance in parental competence;  Improvement in age-appropriate communication |
| Short-term psycho-educational intervention | Hoke, 1997; USA (42) | Description of intervention, Case report | | Model developed for families with parental affective illness (Beardslee et al., 1992) | Improvement in parent’s understanding of own responses to illness, recognizing children’s concerns and understanding of their experiences |
| Struggle for Life Trial | Niemelä et al., 2012; Finland (39) | Evaluation; Quantitative design Baseline, Post intervention ((4,10,18 months after completion), N=19) | | Preventive interventions for mentally ill parents (Solaritaus et al, Beardslee et al.) | Significant decrease in global severity score and psychiatric symptoms profile of SCL-90 at 4 months follow-up in spouses and patients |
| Parent-centered interventions (n=10 studies on n=4 interventions) | | | | | |
| The Enhancing Connection Program | Lewis et al., 2006; USA (50) | | Evaluation (Pilot study): Quantitative design (pre-post, N=13) | Model of coping, contextual model of parenting, Banduras social cognitive theory | *Mothers:* Improvements in functioning (depression, anxiety, self-efficacy), no improvement in mother-child relationship;  *Children:* improvements in total, internalizing and externalizing problems, decrease in cancer-related worries and depressed mood; no improvements in illness-related pressure, disenfranchised grief, anxiety or depressive symptoms (child’s report)  *Qualitative data:* mothers improved to 1) manage own emotions, 2) add to self-care, 3) listen to child, 4) understand child’s behaviour |
|  | Davis Kirsch et al., 2003; USA (51) | | Evaluation (Pilot study): Qualitative design (N=4 families) |  | *Mother:* Preparing the child, practice own skills, being aware of own emotional situation; more open communication, enrichment of mother-child relationship;  *Children:* positive changes in behaviour (patience, dealing with cancer topics), closer relationship with mother |
|  | Brandt et al., 2004; USA (52) | | Implementation (Pilot study) |  | Not investigated |
|  | Lewis et al., 2015; USA (13) | | Evaluation: Quantitative design (IG (n=90) vs. CG (n=86) (baseline, 2 months post, 12 months follow-up)) |  | *Mothers:* Improvements in mothers of IG after 2 months in depression and parenting skills, but not at 12 months; no changes in anxiety, self-efficacy and parenting quality;  *Children*: improvements in IG compared to CG in behavioural and emotional adjustment at 2 months and depressed mood at 2 and 12 months follow-up, no changes in children’s anxiety |
| Art-therapy program for parents | Weiß et al. 2005; Germany (58) | | Description/  Development | Art as support to facilitate communication, clinical practice | Not investigated |
| Being a parent and coping with cancer | Hasson Ohayon & Braun 2011; Israel (53) | | Description/  Development, Feedback | Conclusions from patient and professionals focus groups | Reinforced in chosen to support children, feeling good about parental role, improvement in understanding children’s responses |
| Preventive Intervention for Bereaved Children | Christ et al., 1991; USA (55) | | Description | Clinical experience, literature, parent guidance model | Not investigated |
|  | Christ et al., 2005; USA (56) | | Evaluation: Quantitative design (IG (n=79) vs. CG (n=25) (pre, post, follow-up)) | Clinical experience, literature | No differences in anxiety and depression between groups, but greater decline in IG; high satisfaction with intervention;  children rated parental overall competence and communication competence in IG better than in CG; |
|  | Christ & Siegel, 1991; USA (54) | | Development/ Description of intervention | Clinical experience, literature, parent guidance model | Families described intervention as helpful; reinforced in parental ability to handle children’s reactions and feelings |
|  | Siegel et al., 1990; USA (57) | | Development/ Description of intervention | Clinical experience, clinical and research literature | Not investigated |
| Child-centered interventions (n=7 studies on n=7 interventions) | | | | | |
| The Bear Essential Program | Greening, 1992; USA (49) | | Description/Implementation, Feedback | Erikson's model of developmental stages | Enhanced capacity to deal with crisis, improved communication about cancer topics |
| For kids only | Bedway & Smith, 1997; USA (45) | | Description, Feedback/ Comments | Literature (‘Bear essential’, ‘ Bear Hugs, Butterflies and Turtledovers’), clinical experiences | Alleviated anxiety; created network with other children |
| School-based support group | Call, 1990; USA (46) | | Description and Implementation | clinical experience | Not investigated |
| Quest | Heiney & Lesesne, 1996; USA (47) | | Description, Feedback | Literature based on the “good grieving program” | Information for parents were helpful to improve understanding for children;  Children enjoyed program, more open communication about cancer topics |
| On Belay | Tucker et al., 2013;  USA (16) | | Description, Evaluation: qualitative design (Focus group with parents (n=8) and children (n=12)) | Adventure-based program | Social bond between children, normalize experience, successful master challenges, learn to deal with difficult situations |
| Kids can cope | Taylor-Brown,1993;  Canada (18) | | Description/development | Not stated | Not investigated |
| CLIMB (Children’s lives include moments of bravery) | Semple & McCaughan 2013;  Ireland, UK (48) | | Evaluation: Qualitative design (interviews/focus group (n=4 parents, n=7 children)) | Children are shaped by their environment, behaviour is a function in Social context | Normalization of cancer experience, improved understanding of cancer and coping, improvements in mood and behaviour, potential To create new fears |

IG, Intervention Group; CG, Control Group; QoL, Quality of Life; STEEP, Steps toward effective and enjoyable parenting; SCL-90, Symptom Checklist 90
