# Supplementary material for: Psychosocial Interventions for Families with Parental Cancer and Barriers and Facilitators to Implementation and Use – A Systematic Review
Source: PLoS One. 2016 Jun 8;11(6):e0156967. doi: 10.1371/journal.pone.0156967 (PMC4898703; doi:10.1371/journal.pone.0156967)
Supplement: S5 Table — (DOCX) [file pone.0156967.s006.docx]

S5 Table – *Thematic analysis of barriers and facilitators for using psychosocial support services in families affected by parental cancer*

| Reference | Citation | Category |
| --- | --- | --- |
| Bedway, 1997 | Reported facilitator |  |
|  | The program was changed into one day workshop repeated at selected intervals. Parents have said that because of their busy schedules and drained family energies and time, the one day format is more convenient. | Intervention characteristics/ Structure of the intervention |
|  | Because facilitators are the heart of a successful program, it is essential to develop a core group of them […] Facilitators should enjoy working with children, have excellent communication skills, and be flexible. | Intervention characteristics / Staff with expertise |
|  | Selecting a site for the program can be among the most difficult aspects of program planning […] The site for the “For Kids Only” program is the hospital day care center. | Intervention characteristics/ Accessible location |
|  | Because competition for available funds is fierce, funding sources should be identified early. | Intervention characteristics/ Financial circumstances |
|  | Reported barrier |  |
|  | - |  |
| Brandt et al, 2004 | Reported facilitator |  |
|  | - | - |
|  | Reported barrier |  |
|  | - | - |
| Bugge et al., 2008 | Reported facilitator |  |
|  | Most of the children in our study experienced the situation as unpleasant, and they had questions […] which they did not dare to ask parents | Emotional situation/ Perceived need |
|  | The children’s confidence with project worker was important. | Intervention characteristics / Staff with expertise |
|  | They were provided verbal and written information to take home and discuss with their family | Providing information/ Personal contact with patients |
|  | The three hospitals had shown interest in developing support services for children when their parent had cancer. | Collaboration with institutions/ Motivated staff |
|  | Reported barrier |  |
|  | Four patients were unable to complete the program because of the severity and progress of their illness. | Disease characteristics/ Diagnosis and symptoms |
| Bugge et al., 2009 | Reported Facilitators |  |
|  | Parents joining our Family Support Program certainly wanted help to talk with their children and had felt this need for a long time | Emotional situation/ Perceived need |
|  | […] that parents were inexperienced and profoundly uncomfortable in having to deal with a diagnosis of incurable cancer and had a pressing need for confirmation and reassurance that they had done well. | Emotional situation/ Perceived need |
|  | All family members were invited to participate in the program and the invitation extended to separated or divorced parents and their new partners. | Intervention characteristics/ Structure of the intervention |
|  | It was important that those carrying out the program had a good understanding of patient’s illness situation and how it could affect the family and children. | Intervention characteristics / Staff with expertise |
|  | Make the program available to all patients with poor prognosis and integrate the service into the patient’s mainstream medical and palliative care treatment | Collaboration with institutions/ Part of routine care |
|  | Hospitals should have a contact person who knows what services are available for families and can advise families accordingly. | Collaboration with institutions/ Regular contact and information meetings |
|  | Information about the Program was provided to doctors and nurses in oncology, cancer care and palliative care units within the three hospitals and suggestions offered as to which families could benefit from the program. | Collaboration with institutions/ Regular contact and information meetings |
|  | Reported barriers |  |
|  | […] four patients had to leave the study because of the progress of the illness. | Disease characteristics/ Diagnosis and symptoms |
| Call, 1990 | Reported facilitators |  |
|  | Because of the enthusiastic response by group members and their parents, the agency investigated the possibility of conducting support groups within the school system. | Intervention characteristics/ Accessible location |
|  | Scheduling a group on school ground during school hours eliminates the transportation problems. | Intervention characteristics/ Structure of the intervention |
|  | Reported barriers |  |
|  | […] children often depend on a parent to bring them to agency-based groups. | Organizational difficulties for families/ Distance to intervention location |
|  | The major drawback is the amount of time and effort to collaborate with school personnel. | Collaborations with institutions/ Time and effort for staff |
|  | Because the groups are held during school hours, group members must catch up on any classwork they have missed, take makeup tests and so forth. | Organizational difficulties for families/ Time |
| Christ et al., 2005 | Reported facilitators |  |
|  | The social worker from the patient’s medical unit initially met with the well parent […]. | Providing information/ Personal contact with patients |
|  | The clinicians explained the purpose of the study and the demands of participation. | Providing information/ Proposal by clinician |
|  | They [the team] discussed the patient’s possible eligibility with a research staff supervisor. | Collaboration with institutions/ Regular contact and information meetings |
|  | Reported barriers |  |
|  | Some families moved to another region of the United States around the time the intervention was completed. | Organizational difficulties for families/ Distance to intervention location |
|  | Physical incapacity of ill parent to attend session | Disease characteristics/ Diagnosis and symptoms |
|  | [Other parents] believed that their own and their children’s investment was in the present and the immediate future, not in the past. | Emotional situation/ No need for support |
| Christ et al, 1991 | Reported facilitators |  |
|  | Mothers are more likely to admit their sense of helplessness and respond positively to an offer of emotional support. | Emotional situation/ Perceived need |
|  | Standardized, brief structure of program | Intervention characteristics/ Structure of the intervention |
|  | Fathers who insist that they are already managing well can be moved to participate if they are told, by sharing their experience with us, they can provide information that may help other families. | Emotional situation/ Perceived need |
|  | When recruiting, physicians should emphasize normality of feelings and show responsiveness to parents’ needs | Providing information/ Proposal by clinician |
|  | Rather than asking physicians to identify patients whose life expectancy is approximately six months, we now ask them to identify patients who have a poor prognosis because of advanced disease, but an expected survival time of at least 16 weeks. | Providing information/ Proposal by clinician and  Intervention characteristics/ Structure of the intervention |
|  | Reported barrier |  |
|  | Parents who are denying the progression of the patient’s disease may resist participation because they do not perceive their children’s need for more information, communication and involvement at the time of referral. | Emotional situation/ No need for support |
|  | Physicians worry that we might inadvertently disclose the prognosis of the family before they themselves would feel it propitious; they are concerned that both the family and the staff may feel that they have ‘given up’ on the patient. | Collaboration with institutions/ Mistrust, concerns in intervention |
|  | At times, such families accept the inevitability of the disease progress only later, when the patient becomes more symptomatic and medical deterioration is indisputable. | Disease characteristics/ Phase of disease |
|  | Parents may resist involvement when the patient is very symptomatic and death is perceived as imminent. | Disease characteristics/ Diagnosis and symptoms |
|  | Some patients are simply too ill to participate in a sustained interview. | Disease characteristics/ Diagnosis and symptoms |
|  | Parents may be reluctant that family joins the program because they do not want their children to be confronted with preparation of the advanced disease or death. | Emotional situation/ Emotional overload |
|  | Parents often feel overwhelmed at such times by the stresses of the situation and their own distress. | Emotional situation/ Emotional overload |
|  | Fathers are more likely to feel that the offer of help is an implicit criticism of their current management. They also tend to underestimate the complexity of child rearing and the enormity of their children’s emotional needs at this time. | Emotional situation/ No need for support |
| Christ & Siegel, 1991 | Reported facilitators |  |
|  | We introduced ourselves to the families as interested in doing a study to determine if we could help parents care for their children during a serious recurrence of the cancer. | Providing information/ Personal contact with patients |
|  | We were more successful when we changed the definition of our request for referral of families to: referral when the ill parent’s recurrence is no longer responding to available treatment. | Providing information/ Proposal by clinician and  Intervention characteristics/ Structure of the intervention |
|  | Reported barriers |  |
|  | Oncologist rightly denied their ability to foresee events [parental death] that closely. | Collaboration with institutions/ Mistrust, concerns in intervention |
| Davey et al. 2012 | Reported facilitators |  |
|  | Provided financial help with transportation | Intervention characteristics/ Accessible location |
|  | Financial remuneration after completing sessions | Intervention characteristics/ Incentives |
|  | Refreshments during sessions | Intervention characteristics/ Incentives |
|  | Babysitting for any younger children | Intervention characteristics/ Structure of the intervention |
|  | We trained a spiritually informed African-American female therapist […]. | Intervention characteristics / Staff with expertise  and  Intervention characteristics/ Culturally sensitive |
|  | Taking the time to call parents between sessions [..], involving parents in deciding the best days and times to conduct the sessions | Providing information/ Personal contact with patients |
|  | Perhaps conducting sessions within the African-American community (e.g., the church) and training trusted community leaders […] would have been a better way to overcome difficulties in recruitment | Intervention characteristics/ Accessible location |
|  | We learned that it is important to understand the historical context of African-Americans, partner and build trust with community leaders, and to engage in self-examination as clinical researcher. | Collaboration with institutions/ Regular contact and information meetings |
|  | After several networking meetings the senior pastor invited the first author to attend three of his church services to announce the cancer support group study. | Collaboration with institutions/ Regular contact and information meetings |
|  | We have a close working relationship with an oncology social worker in three of the oncology clinics […]. We also had a close relationship with the nurse navigator who has access to all patients in two of the clinics and additionally met with oncologists at each clinic […]. Each month we met with the support staff and oncologists […]. Our team contacted the clinics every week through telephone and/or emails to remind providers of our study […] | Collaboration with institutions/ Regular contact and information meetings |
|  | Find ways to help busy providers remember to introduce the study to potentially eligible participants | Collaboration with institutions/ Part of routine care |
|  | […] our therapists used a culturally sensitive approach which helped to establish trust, engage, and retain the families. | Intervention characteristics/ Culturally sensitive |
|  | Reported barriers |  |
|  | Even though we partnered with several oncology clinics, the pace of recruitment was much slower than anticipated. Identification of potentially participants by busy staff | Collaboration with institutions/ Time and effort for staff |
|  | The biggest lesson we learned is that all of the strategies we used are not enough to overcome the understandable historical legacy of mistrust in the African-American community. | Emotional situation/ Fear of stigma |
|  | We did not have enough time to build stronger collaborative relations […]. | Collaboration with institutions/ Time and effort for staff |
| Davey et al., 2013 | Reported facilitators |  |
|  | Culturally sensitive approach | Intervention characteristics/ Culturally sensitive |
|  | Conducting sessions within the African-American community (e.g., the church) would have been a better way to overcome difficulties in recruitment | Intervention characteristics/ Accessible location |
|  | Tokens for transportation to each session | Intervention characteristics/ Accessible location |
|  | Babysitting for younger children at home | Intervention characteristics/ Structure of the intervention |
|  | Refreshments | Intervention characteristics/ Incentives |
|  | Financial remuneration after completing each group session | Intervention characteristics/ Incentives |
|  | Reported barriers |  |
|  | Mistrust of the African-American community | Emotional situation/ Fear of stigma |
|  | After realizing that this collaboration was not  as fruitful as expected, we searched for alternative strategies and advertized our study as well as partnered with a local African American mega church. However, these strategiewere not successful in improving the rate of recruitment. | Collaboration with institutions/ Time and effort for staff |
| Davis Kirsch et al., 2003 | Reported facilitators |  |
|  | - |  |
|  | Reported barriers |  |
|  | - |  |
| Dörr et al., 2012 | Reported facilitators |  |
|  | Es empfiehlt sich, betroffene Familien […] gezielt aufzuklären und zu einer präventiven, speziell die Kinder betreffend Beratung zu ermutigen | Providing information/ Personal contact with patients |
|  | Der sorgfältigen Vorbesprechung der Intervention sowie der elterlicher Befürchtungen und ihrer Erfahrungen im psychotherapeutischen Bereich sollte deshalb genügend Raum gegeben werden. | Providing information/ Personal contact with patients |
|  | Reported barriers |  |
|  | Für viele Familien mit Säuglingen und Kleinkindern ist die Inanspruchnahme einer Beratung für diese Altersgruppe ungewohnt und eine große Hürde. | Emotional situation/ Fear of stigma |
|  | Ängste vor Pathologisierung erschweren die Inanspruchnahme | Emotional situation/ Fear of stigma |
|  | Zudem stellt es eine besondere Herausforderung dar, sich unter der eigenen psychischen Belastung auf die Methode der videogestützten Beobachtung einzulassen. | Emotional situation/ Emotional overload |
| Greening, 1992 | Reported facilitators |  |
|  | The social worker in oncological units interviewed the parents to explain the program […]. | Providing information/ Personal contact with patients |
|  | Participants were recruited through referrals by physicians or other staff members | Providing information/ Proposal by clinician |
|  | Reported barriers |  |
|  | It is important to note that newly diagnosed parents did not seem interested in having their children participate in the group. | Disease characteristics/ Phase of disease |
| Hasson-Ohayon et al., 2011 | Reported facilitators |  |
|  | […], we offered patients a one day-workshop [instead of 4 sessions]. | Intervention characteristics/ Structure of the intervention |
|  | Reported barriers |  |
|  | Although they appeared to be very interested they […] had difficulty committing to four intervention sessions. | Organizational difficulties for families/ Time |
|  | […] they seemed overwhelmed by the diagnosis and treatments. | Emotional situation/ Emotional overload |
| Heiney & Lesene, 1996 | Reported facilitators |  |
|  | However to reach more participants, care givers’ awareness of the need for the program needs to be increased. | Emotional situation/ Perceived need |
|  | The program is conducted in the cancer treatment center using conference rooms for group activities and actual treatment areas for tours. | Intervention characteristics/ Accessible location |
|  | The care giver interview before the program begins the process of improving family communication and increasing support to the child […]. Finally, details about the program, such as registration time, mean, and location, are reviewed. | Providing information/ Personal contact with patients |
|  | A direct mail piece goes to all patients diagnosed within the last year. | Providing information/ Personal contact with patients  And  Collaboration with institutions/  Part of routine care |
|  | A direct mail goes to guidance counselors, physicians, hospital staff and mental health professionals. | Collaboration with institutions/ Regular contact and information meeting |
|  | […] direct referral to the program by hospital staff and community mental health professionals is an effective way of promoting the attendance at Quest. | Providing information/ Proposal by clinician |
|  | Reported barriers |  |
|  | […] may be due to the care givers’ inability to assess accurately their children’s emotional adjustment or need for a program. | Emotional situation/ No need for support |
|  | Possibly, once crisis phase is over, care givers do not feel the child is in need for additional support. | Emotional situation/ No need for support  and  Disease characteristics/ Phase of disease |
| Hoke, 1997 | Reported facilitators |  |
|  | - |  |
|  | Reported barriers |  |
|  | - |  |
| John et al., 2010 | Reported facilitators |  |
|  | Mütter, die eine spezielle Unterstützung im Umgang mit ihren Kindern suchen, da sie die Bedürfnisse ihrer Kinder sensibel wahrnehmen oder vielleicht bereits Schwierigkeiten bei ihren Kindern festgestellt haben. | Emotional situation/ Perceived need  And  Emotional situation/ Negative changes in children |
|  | Reported barriers |  |
|  | […], da sie befürchten, dass die neue Umgebung für die Kinder eine zusätzliche Belastung darstellen könnte. | Emotional situation/ Emotional overload |
| John et al., 2013 | Reported facilitators |  |
|  | - |  |
|  | Reported barriers |  |
|  | Affected mothers can apply for participation; approval depends on their health insurance providers. | Organizational difficulties for families/ Health insurance |
| Koch et al., 2013 | Reported facilitators |  |
|  | - |  |
|  | Reported barriers |  |
|  | - |  |
| Kissane et al., 2006 | Reported facilitators |  |
|  | - |  |
|  | Reported barriers |  |
|  | Avoidant families | Emotional situation/ No need for support |
|  | Chaotic/alienated families | Emotional situation/ No need for support |
|  | Patients unwell/died | Disease characteristics/ Diagnosis and symptoms |
|  | Families coping well | Emotional situation/ No need for support |
| Kissane et al., 2007-2008 | Reported facilitators |  |
|  | - |  |
|  | Reported barriers |  |
|  | - | - |
| Komo-Lang et al., 2010 | Reported facilitators |  |
|  | Nach dem ersten Telefonkontakt finden die Gespräche in den Räumen der Psychoonkologie, in Patientenzimmern, oder onkologischen Tageskliniken statt. […] werden auch Gespräche im häuslichen Umfeld angeboten. Kriseninterventionen vor Operationssälen und an Sterbebetten gehören zur täglichen Arbeit. | Intervention characteristics/ accessible location  And  Intervention characteristics/ Structure of the intervention |
|  | Nach einem Telefonkontakt finden die Gespräche […] statt | Providing information/ Personal contact with patients |
|  | Betroffenen Familien erleichtert es den Zugang, wenn onkologisch tätige Ärzte das Beratungsangebot als integrierten und wichtigen Baustein vertreten. | Collaboration with institutions/ Part of routine care  And  Providing information/ Proposal by clinician |
|  | Reported barriers |  |
|  | - |  |
| Kühne et al., 2013 | Reported facilitators |  |
|  | It is important to consider family and disease variables; to adapt to families’ situations and needs. | Intervention characteristics/ Structure of the intervention |
|  | This requires increased flexibility of counselors. | Intervention characteristics/ Structure of the intervention |
|  | The first steps of family recruitment included contacting relevant medical departments; presentations and discussions with medical staff. | Collaboration with institutions/ Regular contact and information meetings |
|  | […] when patients tended to use counseling only if it was introduced by their physician as part of routine care. | Collaboration with institutions/ Part of routine care  And  Providing information/ Proposal by clinician |
|  | […] counseling should be located in the department where the patient is receiving long term treatment. | Intervention characteristics/ Accessible location |
|  | Precise communication was found to be a key issue. To achieve this, it appears advisable to have one specific contact person, to establish good personal working alliances and clarify referral modalities in detail; organizational details should be prepared, address concerns of physicians | Collaboration with institutions/ Regular contact and information meetings |
|  | Reported barriers |  |
|  | Third most common were physician’s concerns e.g. regarding their own resources. | Collaboration with institutions/ Time and effort for staff |
|  | Families’ fears and concerns were mostly related to their fear of stigmatization related to needing mental health services. | Emotional situation/ Fear of stigma |
|  | Sometimes, patients reported to be so preoccupied with their disease that there were no further resources for additional appointments. | Organizational difficulties for families/ Time |
|  | Some partners would only refer acute demand cases […]. Problems with integrating counseling in existing structures meant, for example, problems with establishing contacts with contact persons […] or implementing new procedures as part of liaison partners routines. | Collaboration with institutions/ Mistrust, concerns in intervention |
|  | Physicians’ concerns regarding […] patients’ well-being […] doubts about the benefits of counseling | Collaboration with institutions/ Mistrust, concerns in intervention |
| Lewis et al., 2006 | Reported facilitators |  |
|  | The study team contacted the mother by phone, answered additional questions, and requested that the mother ascertain her child’s interest in participation. | Providing information/ Personal contact with patients |
|  | Site intermediaries approached eligible participants, briefly introduced the study, and pending approval, gave the study team the contact information. | Providing information/ Proposal by clinician |
|  | Reported barriers |  |
|  | - |  |
| Niemelä et al., 2012 | Reported facilitators |  |
|  | The staff treating the patients provided verbal and written information about the study […]. | Providing information/ Proposal by clinician |
|  | Reported barriers |  |
|  | - |  |
| Paschen et al., 2007 | Reported facilitators |  |
|  | Bei der eher kleinen Stichprobe handelt es ich um ein selektiertes Kollektiv von Familien, die eigenmotiviert Unterstützung suchten. | Emotional situation/ Perceived need |
|  | In flexiblen Settings, zu denen neben initialen stützenden Elterngesprächen bedarfsorientiert Einzelsitzungen mit Kindern und Familiengespräche gehören […]. | Intervention characteristics/ Structure of the intervention |
|  | Reported barriers |  |
|  | - |  |
| Romer et al. 2007 | Reported facilitators |  |
|  | Adequate timing depended to some degree on the type of disease that the parent had. | Intervention characteristics/ Structure of the intervention |
|  | Patients only accepted a referral shortly after being diagnosed if they themselves had requested advice on how and when to disclose their diagnosis to their children | Emotional situation/ Parental adjustment to disease |
|  | We interpreted this observation to indicate that patients need enough time to adjust to their new situation themselves and to establish their own coping strategy before they are ready to accept an oﬀer intended to help their children to cope better with the situation. | Emotional situation/ Parental adjustment to disease |
|  | Due to serious condition some patients were not able to take part in sessions. In these cases, a therapist contacted the patients’ spouse by telephone. | Providing information/ Personal contact with patients |
|  | A retrospective analysis of cases in which patients had been approached by a COSIP therapist during a bedside visit after having been referred revealed that families only utilized our counseling intervention when the patient’s physician had personally communicated the option of this referral to the patient. | Providing information/ Proposal by clinician |
|  | […] we and our partners arranged to have child-centered counseling be introduced generally only after a minimum of 3 months following diagnosis, unless patients requested it at an earlier time or prognosis was poor. | Intervention characteristics/ Structure of the intervention |
|  | After we began performing an initial consultation as a standard procedure, the COSIP service was much more readily accepted and utilized by affected families […]. | Collaboration with institutions/ Part of routine care |
|  | After this, patients were contacted by a COSIP therapist during a bedside visit | Providing information/ Personal contact with patients |
|  | All patients with children under 18 years were identified by a research secretary and referred to the COSIP Service. | Collaboration with institutions / Part of routine care |
|  | During implementation period, all patients with children under the age of 18 were identiﬁed by an administrative nurse who was assigned to be the key contact person for the COSIP team. A physician then informed the patients of a scheduled bedside visit by a COSIP therapist. | Collaboration with institutions / Part of routine care  And  Collaboration with institutions/ Regular contact  And  Providing information/ Proposal by clinician |
|  | This liaison was initiated by the head of the Neurosurgical Department, who was interested in integrating family- oriented support for neurosurgical patients. | Collaboration with institutions/ Motivated staff |
|  | The guiding principle of this implementation was to establish easily accessible procedures  for presenting the option of the service to cancer patients within hospital routines. | Collaboration with institutions/ Part of routine care |
|  | The implementation process aimed to sensitize the medical staff to the psychological needs that arise in the children of cancer patients. | Collaborations with institutions/ Regular contact and information meetings |
|  | A COSIP therapist collaborated closely with the psycho-oncological team, which referred all BMT (Bone Marrow Transplant) patients with children under the age of 18. | Collaboration with institutions/ Part of routine care |
|  | Following a presentation of the project within a routine staﬀ meeting, the cooperation was established and included regular participation of a COSIP therapist in weekly staﬀ meetings. | Collaborations with institutions/ information meetings and regular contact |
|  | We concluded from these observations and interpretations that most physicians need to be clinically convinced of the value of the new service in a concrete manner. […] by casuistic evidence illustrating children’s fears and concerns in combination with our concrete therapeutic handling these situations. Such evidence was successfully presented in the form of brief case vignettes that included, for example, children’s drawings | Collaboration with institutions/ Regular contact and information meetings |
|  | Reported barriers |  |
|  | We observed that cancer patients who were referred soon after diagnosis most commonly did not utilize it. | Disease characteristics/ Phase of disease |
|  | We observed that families accepted our interventions far less frequently if we approached them after the beginning of their inpatient phase of the treatment. | Disease characteristics/ Phase of disease |
|  | […] most (patients) acknowledged that they considered the service to certainly be helpful for mostBut declared that they would not need it themselves. | Emotional situation/ No need for support |
|  | Some patients also emphasized the somatic nature of their disease and stated that neither themselves nor their children were in need of psychiatric consultation. | Emotional situation/ No need for support |
|  | We interpreted the reluctance to request such counseling as being the result of a fear of psychiatric stigmatization […]. | Emotional situation/ Fear of stigma |
|  | We interpreted the reluctance to request such counseling as being the result […] of emotional overload. | Emotional situation/ Emotional overload |
|  | Some colleagues felt that our interventions could even be disturbing for their patients’ families by churning up existential fears that had previously been ‘well repressed’. | Collaboration with institutions/ Mistrust, concerns in intervention |
|  | […], we observed in most of our consultation and liaison settings that many physicians were rather hesitant to refer families to our service. | Collaboration with institutions/ Mistrust, concerns in intervention |
|  | Patients utilized the offered counseling in none of the cases in which physicians delegated referral procedures and patient information to other staff members. | Collaboration with institutions/ Time and effort for staff |
|  | Due to the serious condition of most patients in this setting (Neurosurgery) they were most often not able to take part in counseling session | Disease characteristics/ Diagnosis and symptoms |
| Romer et al., 2011 | Reported facilitators |  |
|  | - |  |
|  | Reported barriers |  |
|  | - |  |
| Schmitt et al., 2007 | Reported facilitators |  |
|  | The first author visited the different units of the oncology clinic three times a week […]. From Februar 2003 until the end of the project a nurse […] was employed part time to help with a systematic checking for new patients with children. | Collaborations with institutions/ Regular contact and information meetings |
|  | […], the clininc amended the questionnaire given to each new patient starting his/her treatment. Questions concerning spouse and children were added […], the staff got a clear picture of the family situation of their patients. | Providing information/ Proposal by clinician |
|  | The counseling was offered to all successive new or relapsed cancer patients who had at least one child living with them. | Collaborations with institutions/ Part of routine care |
|  | During their first visit, patients were asked for permission to be contacted personally by family therapist. | Providing information/ Proposal by clinician |
|  | […] information meetings, lectures and presentations were systematically given to the staff of the oncology clinic. | Collaboration with institutions/ Regular contact and information meetings |
|  | The regular attendance of project family therapists in the oncology clinic seemed to be important […]. The presence of family therapists helped the oncology staff members to take cognizance of psychosocial problems of the family in their patients. | Collaboration with institutions/ Regular contact and information meetings |
|  | The situation supports the establishment of a psychosocial team and the integration of child psychiatry as a discipline focusing on children’s mental health in the oncology clinic team. | Collaboration with institutions/ Part of routine care |
|  | Reported barriers |  |
|  | Identification of patients with children was laborious. No information on cancer patients’ families was available. The oncology clinic did not even know if their patients had children or not. | Collaboration with institutions/ Time and effort for staff |
|  | Husband or child against participation | Emotional situation/ No need for support |
|  | Patient too exhausted due to the treatment | Disease characteristics/ Diagnosis and symptoms |
|  | Family living too far away | Organizational difficulties for families/ Distance to intervention location |
|  | No need for psychosocial support | Emotional situation/ No need for support |
|  | Patient already in psychiatric care | Emotional situation/ No need for support |
|  | Prejudices towards psychiatry, and fear of possible stigmatization make it difficult to be perceived as support providers […]. | Emotional situation/ Fear of stigma |
|  | Some families might legitimately baulk at what they see as an intrusion into their efforts to cope with a difficult situation. | Emotional situation/ Emotional overload |
| Semple et al., 2013 | Reported facilitators |  |
|  | Self-referral reflected parents independently seeking professional support through the Cancer Focus Family Support Service. This was often a direct consequence of behavioral or emotional changes in their children. | Emotional situation/ Negative changes in children |
|  | One idea of parents to counteract the timing issue was the provision of a meal for the children […]. | Intervention characteristics/ Structure of the intervention |
|  | The FSW (Family Support Worker) highlighted the potential benefits for these young children in attending this psychosocial intervention. | Providing information/ Personal contact with patients |
|  | Reported barriers |  |
|  | Some parents shared their logistic concerns around the timing of the sessions […]. | Organizational difficulties for families/ Time |
|  | […] families from more rural settings reported on how they were geographically disadvantaged. | Organizational difficulties for families/ Distance to intervention location |
| Siegel et al., 1990 | Reported facilitators |  |
|  | - |  |
|  | Reported barriers |  |
|  | - |  |
| Taylor-Brown et al., 1993 | Reported facilitators |  |
|  | […] provision of transportation by volunteer drivers […]. | Intervention characteristics/ Accessible location |
|  | Reported barriers |  |
|  | About 70% of participants would have been unable to attend the program if transportation would not have been provided. | Organizational difficulties for families/ Distance to intervention location |
|  | […], families of patients at first diagnosis or with good prognosis do not usually refer their children to the program. | Disease characteristics/ Phase of disease |
| Thastum et al., 2006 | Reported facilitators |  |
|  | Our results showed that the parents who agreed to receive counseling showed significantly higher scores on the BDI (Beck Depression Inventory) than the parents of the non-counseling group. […] feelings of depression might motivate to seek family-oriented counseling if it is available. | Emotional situation/ Perceived need |
|  | Reported barriers |  |
|  | - |  |
| Tucker et al., 2013 | Reported facilitators |  |
|  | - |  |
|  | Reported barriers |  |
|  | - |  |
| Weiss et al., 2005 | Reported facilitators |  |
|  | - |  |
|  | Reported barriers |  |
|  | - |  |
| Lewis et al., 2014 | Reported facilitators |  |
|  | After institutional review board approval at the core study center and in each recruitment site, intermediaries contacted potentially eligible other. | Providing information/ Proposal by clinician |
|  | Mothers were then contacted by phone by the study team […]. | Providing information/ Personal contact with patients |
|  | Reported barriers |  |
|  | Too busy to participate […], too much stress | Organizational difficulties for families/ Time |
|  | 50% said they did not want or need the study. | Emotional situation/ No need for support |
|  | […], for example, the mother did not want to tell her children about the breast cancer diagnosis. | Emotional situation/ Emotional overload |
|  | 2% said they were too ill to participate | Disease characteristics/ Diagnosis and symptoms |
