# Supplementary material for: Psychosocial Interventions for Families with Parental Cancer and Barriers and Facilitators to Implementation and Use – A Systematic Review
Source: PLoS One. 2016 Jun 8;11(6):e0156967. doi: 10.1371/journal.pone.0156967 (PMC4898703; doi:10.1371/journal.pone.0156967)
Supplement: S1 Text — (DOCX) [file pone.0156967.s007.docx]

S1 Text: Eletronic database search strategy for EMBASE

EMBASE via Ovid

1. cancer or neoplasm
2. parent* or child or famil*
3. intervention or cousel* or therap*
4. psychosocial or psychol*
5. 1 and 2 and 3 and 4
6. limit 5 to (human and (english or german))
